# Preterm Birth, Family Income, and Intergenerational Income Mobility

**DOI:** 10.1001/jamanetworkopen.2024.15921

**Published:** 2024-06-10

**Authors:** Asma M. Ahmed, Eleanor Pullenayegum, Sarah D. McDonald, Marc Beltempo, Shahirose S. Premji, Roaa Shoukry, Jason D. Pole, Fabiana Bacchini, Prakesh S. Shah, Petros Pechlivanoglou

**Affiliations:** 1 Child Health Evaluative Sciences, The Hospital for Sick Children, Toronto, Ontario, Canada; 2 Department of Epidemiology and Prevention, Wake Forest University School of Medicine, Winston-Salem, North Carolina; 3 Division of Maternal-Fetal Medicine, Department of Obstetrics and Gynecology, McMaster University, Hamilton, Ontario, Canada; 4 Department of Pediatrics, McGill University, Montreal, Quebec, Canada; 5 School of Nursing, Faculty of Health Sciences, Queen’s University, Kingston, Ontario, Canada; 6 Centre for Health Services Research, The University of Queensland, Brisbane, Queensland, Australia; 7 Canadian Premature Babies Foundation, Toronto, Ontario, Canada; 8 Department of Pediatrics, Mount Sinai Hospital, Toronto, Ontario, Canada

## Abstract

**Importance:**

Preterm birth (PTB) has been associated with lower income in adulthood, but associations with intergenerational income mobility and the role of family socioeconomic status (SES) as modifying factor are unclear.

**Objectives:**

To assess whether the association between PTB and income differs according to family SES at birth and to assess the association between PTB and intergenerational income mobility.

**Design, Setting, and Participants:**

This study comprised a matched cohort of live births in Canada between January 1, 1990, and December 31, 1996, with follow-up until December 31, 2018. Statistical analysis was performed between May 2023 and March 2024.

**Exposure:**

Preterm birth, defined as birth between 24 and 37 weeks’ gestational age (with gestational age subcategories of 34-36, 32-33, 28-31, and 24-27 weeks) vs early and full term births (gestational age, 37-41 weeks).

**Main Outcomes and Measures:**

Associations between PTB and annual adulthood income in 2018 Canadian dollars were assessed overall (current exhange rate: $1 = CAD $1.37) and stratified by family income quintiles, using generalized estimating equation regression models. Associations between PTB and percentile rank change (ie, difference between the rank of individuals and their parents in the income distribution within their respective generations) and upward or downward mobility (based on income quintile) were assessed using linear and multinomial logistic regressions, respectively.

**Results:**

Of 1.6 million included births (51.1% boys and 48.9% girls), 6.9% infants were born preterm (5.4% born at 34-36 weeks, 0.7% born at 32-33 weeks, 0.5% born at 28-31 weeks, and 0.2% born at 24-27 weeks). After matching on baseline characteristics (eg, sex, province of birth, and parental demographics) and adjusting for age and period effects, PTB was associated with lower annual income (mean difference, CAD −$687 [95% CI, −$788 to −$586]; 3% lower per year), and the differences were greater among those belonging to families in the lowest family SES quintile (mean difference, CAD −$807 [95% CI, −$998 to −$617]; 5% lower per year). Preterm birth was also associated with lower upward mobility and higher downward mobility, particularly for those born earlier than 31 weeks’ gestational age (24-27 weeks: mean difference in percentile rank change, −8.7 percentile points [95% CI, −10.5 to −6.8 percentile points]).

**Conclusions and Relevance:**

In this population-based matched cohort study, PTB was associated with lower adulthood income, lower upward social mobility, and higher downward mobility, with greater differences among those belonging to economically disadvantaged families. Interventions to optimize socioeconomic outcomes of preterm-born individuals would need to define target population considering SES.

## Key Points

**Question:**

Is preterm birth (PTB) associated with adulthood income and intergenerational income mobility?

**Findings:**

In this population-based matched cohort study of 1.6 million births, PTB was associated with lower yearly income, decreased upward mobility, and increased downward mobility during adulthood, with more pronounced differences in families with low socioeconomic status. These differences varied by gestational age at birth, with increasing differences as gestational age decreased.

**Meaning:**

This study suggests that PTB was associated with lower income during adulthood and less potential for social mobility, particularly for individuals from families with low socioeconomic status.

## Teaser

This cohort study assesses whether the association between preterm birth and adulthood income differs according to family socioeconomic status at birth and assesses the association between preterm birth and intergenerational income mobility.

## Introduction

Preterm birth (PTB; birth before 37 weeks of gestation) affects approximately 10% of births worldwide and is a leading cause of perinatal mortality and morbidity.^1,2^ Preterm birth is associated with adverse health outcomes over the life course,^3,4,5^ including respiratory complications, cognitive impairment, and mental and behavioral problems.^4,6,7,8^ In addition to long-term health consequences,^9,10,11^ individuals born preterm experience lower educational attainment, income, and employment rates during adulthood than their term-born counterparts.^12,13,14^

Low family socioeconomic status (SES) is a recognized risk factor for impaired childhood cognitive development, school performance, and socioeconomic outcomes during adulthood.^15,16,17,18^ Although previous studies have examined how family SES modifies the association of PTB with cognitive and educational outcomes,^19,20,21,22^ no study, to our knowledge, has assessed whether families’ low SES exacerbates the association of PTB with economic outcomes. Studying the long-term effect of PTB across socioeconomic groups might help identify groups that would benefit from targeted interventions.

In an era of increasing inequality worldwide,^23,24^ it is important to examine the intergenerational transmission of SES to provide a richer picture of socioeconomic inequality.^25^ Studies have shown that intergenerational mobility—how children change places within the socioeconomic distribution relative to their parents—has decreased over time in several countries, including Canada.^24,26,27^ The evidence of the link between health in early life—a key factor associated with lifelong chances and economic opportunities—and intergenerational social mobility is scarce.^12,13,14,28,29,30,31,32,33,34,35,36^ One ecological study has documented a negative association between the incidence of low-weight births and intergenerational economic mobility at a county level.^35^ Nevertheless, it is hard to draw conclusions on individual-level associations based on these results due to the ecological fallacy bias (ie, making inferences about individuals based on group-level associations).^37^ Another study has found that those born late preterm had lower occupation status, income, and education than their parents, compared with full term–born individuals.^14^ This study, however, focused only on late PTB and did not consider the whole spectrum of PTBs. In the present study, we expanded on the existing literature and used nationally representative, family-linked data to (1) assess whether the associations between PTB and child income in adulthood differ according to family income at birth and (2) examine the association between PTB and intergenerational social mobility and the potential effect modification by family income at birth.

## Methods

### Settings

Using a secure environment for linking databases at Statistics Canada, the Social Data Linkage Environment, we linked population-level health, demographic, and income data across generations, resulting in rich, family-linked dataset spanning more than 28 years. We created a national cohort of all individuals born between January 1, 1990, and December 31, 1996, and their families and tracked them until December 31, 2018 (aged 22-28 years). We identified births from the Canadian Vital Statistics–Birth database (CVSB), which uniquely links maternal and child birth records. We subsequently linked the CVSB to income tax files and vital statistics using unique personal identifiers (further details about datasets in eTable 1 in Supplement 1). Data privacy and confidentiality were maintained throughout the data linkage and use. We accessed deidentified data only through a secure server at Statistics Canada. The cohort study was approved by the Hospital for Sick Children Research Ethics Board and Statistics Canada with a waiver of participant consent because retrospective deidentified data were used. We followed the Strengthening the Reporting of Observational Studies in Epidemiology (STROBE) reporting guideline.^38^

### Exposure

Information about gestational age (GA) at birth in completed weeks was obtained from the CVSB. Gestational age was derived from birth certificates that were completed by parents and health care professionals. Preterm birth was defined as birth before 37 completed weeks of gestation, and early and full term birth (the reference group; hereafter, full term) was defined as birth at 37 to 41 weeks of gestation. Preterm birth was also further categorized as extremely preterm (22-27 weeks), very preterm (28-31 weeks), moderately preterm (32-33 weeks), and late preterm (34-36 weeks).

### Matching

Our matching variables included individuals’ sex, birth plurality, province of birth, birth year, parental age at birth, maternal marital status, maternal parity, and parental place of birth based on Statistical Classification of Countries and Areas of Interest for Social Statistics, obtained from the CVSB.^39^ We also included family income quintiles at birth (quintiles range from 1 to 5, with 1 as the lowest income quintile and 5 as the highest) and maternal place of residence at the time of delivery (rural or urban) obtained from the tax files (eTable 1 in Supplement 1).

To address measured confounding, we used coarsened exact matching to match individuals born preterm to those born at term on baseline covariates.^40^ We created multiple strata representing unique combinations of covariate categories and subsequently excluded individuals in strata with no preterm or term-born individuals. The matching procedure resulted in strata with varying numbers of preterm individuals and term controls; therefore, weights were created and used in all subsequent analyses to account for the differential strata sizes. We performed separate matching for GA subcategories.

### Family Income at Birth

Family income was defined as the mean annual family pretax income during the birth year and the preceding year, including income of both parents if present, obtained from maternal tax return files. The family income included the sum of the household’s annual income from employment (wages, salaries, and commissions), self-employment income, interest and investment income, income from government programs (eg, child benefits, social assistance, and parental leave benefits), and tax credits and deductions (eg, disability tax credits and pension contributions).^41^ Family income was adjusted for family size by dividing the family income by the square root of family size.^42^

### Child Income in Adulthood

Personal income when an individual turned 18 years was defined as the annual pretax income from all sources (employment income, social benefits, and tax credits). Child income was adjusted to 2018 Canadian dollars (current exhange rate: $1 = CAD $1.37) using the national level Consumer Price Index to obtain a consistent measure of income over time.^43^

### Intergenerational Income Mobility

Intergenerational income mobility was estimated using percentile rank change, calculated as the arithmetic difference between the rank of individuals and their parents in the income distribution within their respective generations.^24^ Therefore, upward mobility indicates that a child’s rank in the income distribution during adulthood is higher than their parents’ rank in the prior generation. Family income at baseline was converted into percentiles, calculated separately for each birth year and maternal age category. Child income percentiles were calculated according to child income at the end of follow-up (aged 22-28 years) and were computed separately for each calendar year and age in years. We chose to use percentile rank changes because they are less affected by life-cycle bias (income is observed only within a limited period of the life cycle) and more robust to the presence of low incomes and different model specifications than the absolute difference in income.^44^

We also measured intergenerational income mobility using a transition matrix, a matrix of probabilities that the individual’s income observed in a specific quintile will be consistent with their parents’ quintile.^23^ Family income was converted into quintiles (estimated separately by birth year and maternal age), and child income quintiles were calculated according to their income at the end of follow-up (estimated separately by calendar year and age in years). Individuals were categorized into 3 groups according to whether they moved up, down, or stayed in the same quintile relative to their parent.

### Statistical Analysis

Statistical analysis was performed between May 2023 and March 2024. For aim 1 (child income as outcome), we estimated mean differences in child income between groups using generalized estimating equation linear regression models (to account for repeated measures of income), overall and stratified by family income quintiles.^45^ We also examined differences in mean income on the log scale to account for the skewness of income distribution.^46^ We adjusted generalized estimating equation models for age and calendar year modeled flexibly using restricted cubic splines to account for age and period effects.^47,48^ For intergenerational income mobility, we used linear regression models to estimate the associations between PTB and percentile rank change. For the quintile transition matrix, we estimated the probabilities of transitioning to each of the 5 child income quintiles, conditional on family income quintiles. We also calculated risk ratios for moving up or down, relative to staying in the same quintile as their parent, using multinomial logistic regressions. All analyses were conducted using both binary PTB variable and GA categories. We used multiple imputations by chained equations (5 imputations) for missing income in some years (approximately 6%).^49^ We then estimated all models across the 5 imputed datasets and pooled the results. In the main analyses, we excluded all years after an individual’s death (16 940 participants died during the follow-up).

In secondary analyses, we repeated all analyses using child family income as the measure of child income during adulthood.^24^ We also reran all analyses and assigned individuals who died during the follow-up zero income to prevent selection bias introduced from differential losses to follow-up, as preterm-born individuals had higher mortality. To investigate the effect of missing data due to the lack of linkage to maternal tax records or missing all child tax records, we fitted models with inverse probability of censoring weighting. Furthermore, we repeated all analyses only among singletons and conducted sex-stratified analyses to assess potential differences by sex. For analyses with percentile rank change as the outcome, we ran stratified analyses by family income quintiles at baseline and age. To check the robustness of our findings with respect to other measures of mobility, we used the difference between the child and family income *z* scores as the outcome, the change in income *z* score. Income *z* scores represent standardized measures of income relative to a reference population, in this case, all individuals of the same age within a given calendar year.^50^ Precision around point estimates was provided using 2-sided 95% CIs, and estimates were deemed statistically significant if the 95% CIs did not include the null value. We used R, version 4.2.1 (R Project for Statistical Computing) for descriptive analyses, matching, and multiple imputations and Stata, version 17 (StataCorp LLC) for generalized estimating equation and multinomial regressions.

## Results

### Study Population

**Figure 1.**
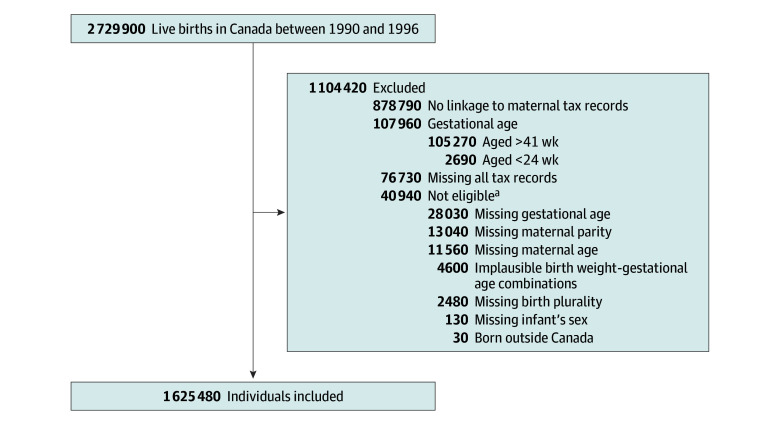
Flowchart Illustrating the Formation of the Study Cohorts ^a^Numbers total more than 40 940 because there is overlap between different missing categories.

We identified 2 729 400 live births occurring between 1990 and 1996. We excluded 1.3% of births with missing baseline characteristics, 0.2% with birth weight for GA *z* score more than 4 SD from the mean,^51^ and 4.0% with GA less than 24 weeks or more than 41 weeks. We subsequently excluded 878 790 births (34.0%) with no linkage to maternal tax records at baseline and 76 730 individuals (3.0%) who did not have any tax records during the entire follow-up period (Figure 1). Poor linkage to maternal tax records at baseline was more likely to occur in earlier birth cohorts (>85.1% of excluded births occurred in 1990-1992). Individuals with no tax record during the entire follow-up period were more likely to be born preterm or of multiple births; born in the province of Quebec; born to young (<20 years), single, or multiparous (≥4 previous live births) mothers, parents born outside Canada, or families with the lowest income quintile at baseline; and to have missing data on father’s age (eTable 2 in Supplement 1).

The full unmatched cohort included 1.6 million births (51.1% boys and 48.9% girls) (Table 1). The rate of PTB in the final study population was 6.9% (5.4% born at 34-36 weeks, 0.7% born at 32-33 weeks, 0.5% born at 28-31 weeks, and 0.2% born at 24-27 weeks). Male infants and twins or higher-order multiples were more likely to be born preterm, as were births to mothers younger than 20 years or aged 35 years or older, single mothers or mothers with missing data on marital status, multiparous mothers, fathers whose age was 40 years or more or missing, and families with parents born outside Canada and families with low SES (Table 1 and eTable 3 in Supplement 1). The matched cohort included 974 930 births (88 320 preterm and 886 600 full term). Differences in the distribution of characteristics by PTB were eliminated in the matched cohort (Table 1 and eTable 3 in Supplement 1).

**Table 1.** Characteristics of Preterm and Term-Born Individuals in the Unmatched and Matched Cohorts, Canada, 1990-1996 Births^a^

Characteristic	Unmatched cohort (N = 1 625 480), No. (%)			Matched cohort (n = 974 930), No. (%) (weighted)	
	Early and full term (n = 1 513 670)	Preterm (n = 111 820)	SMD	Early and full term (n = 886 600)	Preterm (n = 88 320)
Individual’s sex					
Female	743 140 (49.1)	51 400 (46.0)	0.06	403 970 (45.6)	40 240 (45.6)
Male	770 530 (50.9)	60 410 (54.0)		482 640 (54.4)	48 080 (54.4)
Birth plurality					
Singleton	1 497 750 (98.9)	97 040 (86.8)	0.49	847 250 (95.6)	84 400 (95.6)
Multiple	15 920 (1.1)	14 780 (13.2)		39 350 (4.4)	3920 (4.4)
Maternal parity					
0	712 990 (47.1)	54 510 (48.7)	0.09	469 180 (52.9)	46 740 (52.9)
1	511 330 (33.8)	34 080 (30.5)		267 440 (30.2)	26 640 (30.2)
2	197 460 (13.0)	14 850 (13.3)		104 700 (11.8)	10 430 (11.8)
3	60 190 (4.0)	5220 (4.7)		29 470 (3.3)	2940 (3.3)
≥4	31 710 (2.1)	3170 (2.8)		15 810 (1.8)	1580 (1.8)
Maternal age, y					
<20	79 080 (5.2)	6910 (6.2)	0.11	55 190 (6.2)	5500 (6.2)
20-24	301 690 (19.9)	22 280 (19.9)		178 850 (20.2)	17 820 (20.2)
25-29	551 210 (36.4)	37 230 (33.3)		309 590 (34.9)	30 840 (34.9)
30-34	430 810 (28.5)	31 340 (28.0)		252 150 (28.4)	25 120 (28.4)
35-39	133 830 (8.8)	12 130 (10.8)		82 770 (9.3)	8250 (9.3)
≥40	17 060 (1.1)	1920 (1.7)		8050 (0.9)	800 (0.9)
Paternal age, y					
<25	170 260 (11.2)	13 300 (11.9)	0.10	106 220 (12.0)	10 580 (12.0)
25-29	438 380 (29.0)	30 170 (27.0)		250 150 (28.2)	24 920 (28.2)
30-34	496 670 (32.8)	34 430 (30.8)		285 330 (32.2)	28 420 (32.2)
35-39	230 290 (15.2)	17 520 (15.7)		132 180 (14.9)	13 170 (14.9)
≥40	92 060 (6.1)	8160 (7.3)		62 640 (7.1)	6240 (7.1)
Missing	86 020 (5.7)	8240 (7.4)		50 080 (5.6)	4990 (5.6)
Maternal place of birth					
Africa	10 620 (0.7)	980 (0.9)	0.17	3920 (0.4)	390 (0.4)
Asia	83 150 (5.5)	8350 (7.5)		61 640 (7.0)	6140 (7.0)
Canada	1 234 900 (81.6)	85 080 (76.1)		707 300 (79.8)	70 460 (79.8)
Central and South America	24 010 (1.6)	2560 (2.3)		13 510 (1.5)	1350 (1.5)
Europe	68 880 (4.6)	4930 (4.4)		27 530 (3.1)	2740 (3.1)
North America, excluding Canada	17 550 (1.2)	1080 (1.0)		2900 (0.3)	290 (0.3)
Other	74 560 (4.9)	8850 (7.9)		69 790 (7.9)	6950 (7.9)
Paternal place of birth					
Africa	12 620 (0.8)	1060 (0.9)	0.17	4300 (0.5)	430 (0.5)
Asia	80 590 (5.3)	7850 (7.0)		59 210 (6.7)	5900 (6.7)
Canada	1 145 150 (75.7)	77 580 (69.4)		647 770 (73.1)	64 530 (73.1)
Central and South America	24 750 (1.6)	2500 (2.2)		13 150 (1.5)	1310 (1.5)
Europe	79 420 (5.2)	5510 (4.9)		32 190 (3.6)	3210 (3.6)
North America, excluding Canada	14 300 (0.9)	920 (0.8)		2360 (0.3)	240 (0.3)
Other	156 840 (10.4)	16 400 (14.7)		127 630 (14.4)	12 710 (14.4)
Maternal marital status at birth					
Married	1 034 400 (68.3)	70 240 (62.8)	0.14	573 960 (64.7)	57 180 (64.7)
Other	81 900 (5.4)	9260 (8.3)		62 730 (7.1)	6250 (7.1)
Missing	27 470 (1.8)	2410 (2.2)		9350 (1.1)	930 (1.1)
Single	369 900 (24.4)	29 910 (26.7)		240 560 (27.1)	23 970 (27.1)
Province of birth					
Alberta	169 490 (11.2)	12 350 (11.0)	0.10	92 670 (10.5)	9230 (10.5)
Atlantic provinces	112 730 (7.4)	8160 (7.3)		64 010 (7.2)	6380 (7.2)
British Columbia	188 710 (12.5)	12 280 (11.0)		87 290 (9.8)	8700 (9.8)
Manitoba	69 820 (4.6)	5390 (4.8)		37 410 (4.2)	3730 (4.2)
Ontario	530 330 (35.0)	44 050 (39.4)		364 560 (41.1)	36 320 (41.1)
Quebec	375 020 (24.8)	25 060 (22.4)		209 120 (23.6)	20 830 (23.6)
Saskatchewan	61 340 (4.1)	4200 (3.8)		30 190 (3.4)	3010 (3.4)
Yukon, Nunavut, and Northwest Territory	6230 (0.4)	340 (0.3)		1350 (0.2)	130 (0.2)
Birth year					
1990	92 550 (6.1)	4900 (4.4)	0.14	38 620 (4.4)	3850 (4.4)
1991	120 180 (7.9)	7000 (6.3)		55 910 (6.3)	5570 (6.3)
1992	131 420 (8.7)	7800 (7.0)		62 890 (7.1)	6270 (7.1)
1993	293 840 (19.4)	21 570 (19.3)		172 290 (19.4)	17 160 (19.4)
1994	299 070 (19.8)	23 020 (20.6)		182 780 (20.6)	18 210 (20.6)
1995	292 950 (19.4)	23 860 (21.3)		189 120 (21.3)	18 840 (21.3)
1996	283 660 (18.7)	23 660 (21.2)		185 000 (20.9)	18 430 (20.9)
Maternal place of residence					
Urban	1 134 640 (75.0)	86 520 (77.4)	0.06	692 820 (78.1)	69 020 (78.1)
Rural	379 020 (25.0)	25 300 (22.6)		193 790 (21.9)	19 310 (21.9)
Family income quintile at baseline					
1 (Lowest)	286 850 (19.0)	25 880 (23.1)	0.12	194 770 (22.0)	19 400 (22.0)
2	301 380 (19.9)	23 570 (21.1)		182 120 (20.5)	18 140 (20.5)
3	307 230 (20.3)	21 450 (19.2)		170 560 (19.2)	16 990 (19.2)
4	309 770 (20.5)	20 720 (18.5)		169 270 (19.1)	16 860 (19.1)
5 (Highest)	308 450 (20.4)	20 200 (18.1)		169 880 (19.2)	16 920 (19.2)

Abbreviation: SMD, standardized mean difference.

^a^ All numbers were rounded to the nearest 10 for confidentiality reasons; all SMDs were less than 0.001 after matching.

### PTB and Child Income in Adulthood

Individuals were followed up until the end of 2018 for a median of 24 years (IQR, 23-25 years) to ascertain economic outcomes. The mean (SD) total income from all sources for children per year in the unmatched cohort was CAD $21 200 ($31 400) (CAD $21 300 [$24 100] for full term and CAD $19 700 [$8500] for preterm-born individuals).

Table 2 presents associations between PTB and child total income from all sources during adulthood, overall and stratified by family income quintiles at baseline. The income of individuals born preterm was CAD $687 lower per year (95% CI, −$788 to −$586), 3% lower per year, than those born at full term in the matched cohort, and the differences were greater with lower GA. Absolute and relative differences in mean income differed slightly according to family’s income quintile, with greater differences in quintiles 1 to 3 (Q1: mean difference, CAD −$807 [95% CI, −$998 to −$617]; 5% lower per year) and lesser differences in quintiles 4 and 5 (Table 2). The results presented in eTable 5 in Supplement 1 indicate a negative association of PTB with percentile change for all GA categories across family quintile groups. More specifically, for those born at 34 to 36 weeks’ GA, higher family income quantiles at birth (quintiles 4 and 5) decreased the association between PTB and annual income, although this interaction was not statistically significant across other GA categories.

**Table 2.** Associations Between Preterm Birth and Annual Income at or After the Age of 18 Years for Individuals Born in Canada, 1990-1996, in Comparison With Term-Born Children^a^

Category	Overall	Family quintile 1 (lowest)	Family quintile 2	Family quintile 3	Family quintile 4	Family quintile 5 (highest)
**Unmatched cohort, mean annual employment income difference, CAD$ (95% CI)** ^b^						
Preterm (<37 wk)	−1579 (−1663 to −1495)	−1559 (−1710 to −1409)	−1838 (−2005 to −1670)	−1406 (−1591 to −1220)	−1006 (−1201 to −812)	−841 (−1074 to −608)
Gestational age category, wk						
34-36	−1349 (−1443 to −1255)	−1405 (−1575 to −1235)	−1631 (−1817 to −1445)	−1187 (−1393 to −980)	−784 (−1001 to −568)	−490 (−749 to −232)
32-33	−1670 (−1921 to −1419)	−1605 (−2043 to −1167)	−1818 (−2325 to −1311)	−1820 (−2371 to −1269)	−955 (−1530 to −380)	−1016 (−1732 to −300)
28-31	−2687 (−2967 to −2408)	−2206 (−2690 to −1722)	−2912 (−3493 to −2331)	−1981 (−2610 to −1352)	−1989 (−2667 to −1311)	−3109 (−3859 to −2360)
24-27	−5427 (−5859 to −4996)	−4336 (−5078 to −3593)	−5221 (−6030 to −4413)	−5478 (−6429 to −4528)	−5521 (−6563 to −4480)	−5308 (−6647 to −3970)
**Matched cohort model 1: mean annual employment income difference, CAD$ (95% CI)** ^c^						
Preterm (<37 wk)	−697 (−801 to −592)	−814 (−1011 to −618)	−786 (−993 to −579)	−786 (−1011 to −560)	−538 (−773 to −303)	−545 (−830 to −259)
Gestational age category, wk						
34-36	−459 (−573 to −345)	−621 (−840 to −402)	−569 (−796 to −341)	−527 (−773 to −280)	−302 (−559 to −45)	−253 (−559 to 54)
32-33	−902 (−1231 to −573)	−952 (−1557 to −347)	−853 (−1528 to −178)	−1157 (−1865 to −448)	−694 (−1408 to 19)	−898 (−1840 to 44)
28-31	−2001 (−2362 to −1640)	−1717 (−2364 to −1071)	−2165 (−2911 to −1419)	−1951 (−2754 to −1149)	−1582 (−2475 to −688)	−2658 (−3585 to −1730)
24-27	−4481 (−5055 to −3908)	−3902 (−4947 to −2858)	−3979 (−5014 to −2944)	−5177 (−6501 to −3854)	−5312 (−6554 to −4069)	−3954 (−5787 to −2121)
**Matched cohort model 2: mean annual employment income difference, CAD$ (95% CI)** ^c^ ^,^ ^d^						
Preterm (<37 wk)	−687 (−788 to −586)	−807 (−998 to −617)	−775 (−975 to −576)	−776 (−993 to −559)	−531 (−758 to −305)	−528 (−806 to −249)
Gestational age category, wk						
34-36	−455 (−566 to −345)	−617 (−829 to −405)	−565 (−784 to −346)	−526 (−763 to −289)	−300 (−547 to −52)	−246 (−544 to 53)
32-33	−887 (−1208 to −565)	−948 (−1540 to −356)	−825 (−1482 to −168)	−1145 (−1821 to −469)	−684 (−1373 to 5)	−875 (−1802 to 52)
28-31	−1950 (−2303 to −1597)	−1716 (−2345 to −1086)	−2121 (−2848 to −1394)	−1865 (−2644 to −1086)	−1491 (−2370 to −612)	−2590 (−3490 to −1689)
24-27	−4278 (−4835 to −3721)	−3775 (−4779 to −2771)	−3833 (−4846 to −2820)	−4864 (−6136 to −3591)	−5196 (−6415 to −3978)	−3525 (−5293 to −1758)
**Unmatched cohort, ratio of income per year (95% CI)** ^b^						
Preterm (<37 wk)	0.92 (0.92 to 0.93)	0.91 (0.90 to 0.92)	0.91 (0.90 to 0.92)	0.93 (0.92 to 0.94)	0.95 (0.94 to 0.96)	0.96 (0.95 to 0.97)
Gestational age category, wk						
34-36	0.93 (0.93 to 0.94)	0.92 (0.91 to 0.93)	0.92 (0.91 to 0.93)	0.94 (0.93 to 0.95)	0.96 (0.95 to 0.97)	0.98 (0.97 to 0.99)
32-33	0.92 (0.91 to 0.93)	0.91 (0.88 to 0.93)	0.91 (0.88 to 0.93)	0.91 (0.89 to 0.94)	0.96 (0.93 to 0.98)	0.96 (0.93 to 0.99)
28-31	0.87 (0.86 to 0.88)	0.87 (0.85 to 0.90)	0.85 (0.83 to 0.88)	0.91 (0.88 to 0.94)	0.91 (0.88 to 0.94)	0.87 (0.83 to 0.90)
24-27	0.74 (0.72 to 0.76)	0.75 (0.71 to 0.79)	0.74 (0.70 to 0.78)	0.74 (0.70 to 0.79)	0.74 (0.70 to 0.79)	0.77 (0.71 to 0.83)
**Matched cohort model 1: ratio of income per year (95% CI)** ^c^						
Preterm (<37 wk)	0.96 (0.96 to 0.97)	0.95 (0.94 to 0.96)	0.96 (0.95 to 0.97)	0.96 (0.95 to 0.97)	0.97 (0.96 to 0.99)	0.98 (0.96 to 0.99)
Gestational age category, wk						
34-36	0.98 (0.97 to 0.98)	0.96 (0.95 to 0.98)	0.97 (0.96 to 0.98)	0.97 (0.96 to 0.99)	0.99 (0.97 to 1.00)	0.99 (0.98 to 1.00)
32-33	0.96 (0.94 to 0.97)	0.94 (0.91 to 0.98)	0.96 (0.92 to 0.99)	0.94 (0.91 to 0.98)	0.97 (0.93 to 1.00)	0.96 (0.92 to 1.00)
28-31	0.90 (0.88 to 0.92)	0.90 (0.86 to 0.94)	0.89 (0.85 to 0.93)	0.91 (0.87 to 0.95)	0.93 (0.89 to 0.97)	0.88 (0.84 to 0.92)
24-27	0.77 (0.75 to 0.80)	0.77 (0.72 to 0.83)	0.79 (0.74 to 0.84)	0.75 (0.70 to 0.82)	0.75 (0.69 to 0.80)	0.82 (0.75 to 0.91)
**Matched cohort model 2: ratio of income per year (95% CI)** ^c^ ^,^ ^d^						
Preterm (<37 wk)	0.97 (0.96 to 0.97)	0.95 (0.94 to 0.97)	0.96 (0.94 to 0.97)	0.96 (0.95 to 0.98)	0.97 (0.96 to 0.99)	0.99 (0.97 to 1.00)
Gestational age category, wk						
34-36	0.98 (0.97 to 0.98)	0.96 (0.95 to 0.98)	0.97 (0.95 to 0.98)	0.97 (0.96 to 0.99)	0.98 (0.97 to 1.00)	1.00 (0.98 to 1.01)
32-33	0.96 (0.94 to 0.98)	0.95 (0.91 to 0.99)	0.94 (0.90 to 0.98)	0.96 (0.91 to 1.00)	0.97 (0.93 to 1.01)	0.97 (0.93 to 1.03)
28-31	0.90 (0.88 to 0.92)	0.91 (0.86 to 0.96)	0.88 (0.84 to 0.92)	0.90 (0.86 to 0.95)	0.90 (0.86 to 0.95)	0.89 (0.84 to 0.94)
24-27	0.80 (0.77 to 0.84)	0.80 (0.73 to 0.87)	0.79 (0.72 to 0.86)	0.81 (0.73 to 0.89)	0.76 (0.69 to 0.83)	0.89 (0.80 to 0.99)

^a^ Current exhange rate: $1 = CAD $1.37.

^b^ For 1 614 150 individuals; 11 727 110 person-years.

^c^ For 968 550 individuals with gestational age less than 37 weeks (6 966 590 person-years); for 897 970 individuals with gestational age 34 to 36 weeks (6 465 520 person-years); for 315 050 individuals with gestational age 32 to 33 weeks (2 207 450 person-years); for 229 770 individuals with gestational age 28 to 31 weeks (1 611 600 person-years); for 103 160 individuals with gestational age 24 to 27 weeks (712 350 person-years).

^d^ Model 2 used the matched sample and further adjusted for the calendar year and age modeled using restricted cubic splines.

### Intergenerational Income Mobility

Table 3 shows the mean percentile rank change and mean differences in the percentile rank change by PTB (and GA subcategories) in the unmatched and matched cohorts. Results in the matched cohort showed that PTB was associated with minimal downward rank mobility (mean difference in percentile rank change, −1.24 [95% CI, −1.54 to −0.94]), with more noticeable differences in the lowest GA categories (mean difference, −8.7 points [95% CI, −10.5 to −6.8 points] for those born at 24-27 weeks’ GA).

**Table 3.** Associations Between Preterm Birth and Mean Difference in Percentile Rank Change for Individuals Born in Canada, 1990-1996

Category	Unmatched cohorts^a^		Matched cohorts^b^	
	Mean percentile rank change (95% CI)	Mean difference in percentile rank change (95% CI)	Mean percentile rank change (95% CI)	Mean difference in percentile rank change (95% CI)
Preterm (<37 wk)	−0.07 (−0.30 to 0.17)	1.07 (0.83 to 1.31)	−0.96 (−1.22 to −0.70)	−1.24 (−1.54 to −0.94)
Early and full term (37-41 wk)	−1.14 (−1.20 to −1.07)	[Reference]	0.28 (0.20 to 0.36)	[Reference]
Gestational age category, wk				
34-36	0.41 (0.13 to 0.69)	1.55 (1.27 to 1.83)	−0.53 (−0.82 to −0.23)	−0.71 (−1.04 to −0.38)
32-33	−0.73 (−1.42 to −0.04)	0.40 (−0.29 to 1.10)	−1.67 (−2.48 to −0.87)	−1.98 (−2.87 to −1.09)
28-31	−1.91 (−2.74 to −1.08)	−0.77 (−1.61 to 0.06)	−3.17 (−4.14 to −2.20)	−3.83 (−4.92 to −2.74)
24-27	−7.24 (−8.66 to −5.82)	−6.1 (−7.52 to −4.68)	−6.80 (−8.45 to −5.15)	−8.65 (−10.46 to −6.83)

^a^ For 1 614 150 individuals.

^b^ For 968 550 individuals with gestational age less than 37 weeks category; for 897 970 individuals with gestational age 34 to 36 weeks; for 315 050 individuals with gestational age 32 to 33 weeks; for 229 770 individuals with gestational age 28 to 31 weeks; and for 103 160 individuals with gestational age 24 to 27 weeks.

**Figure 2.**
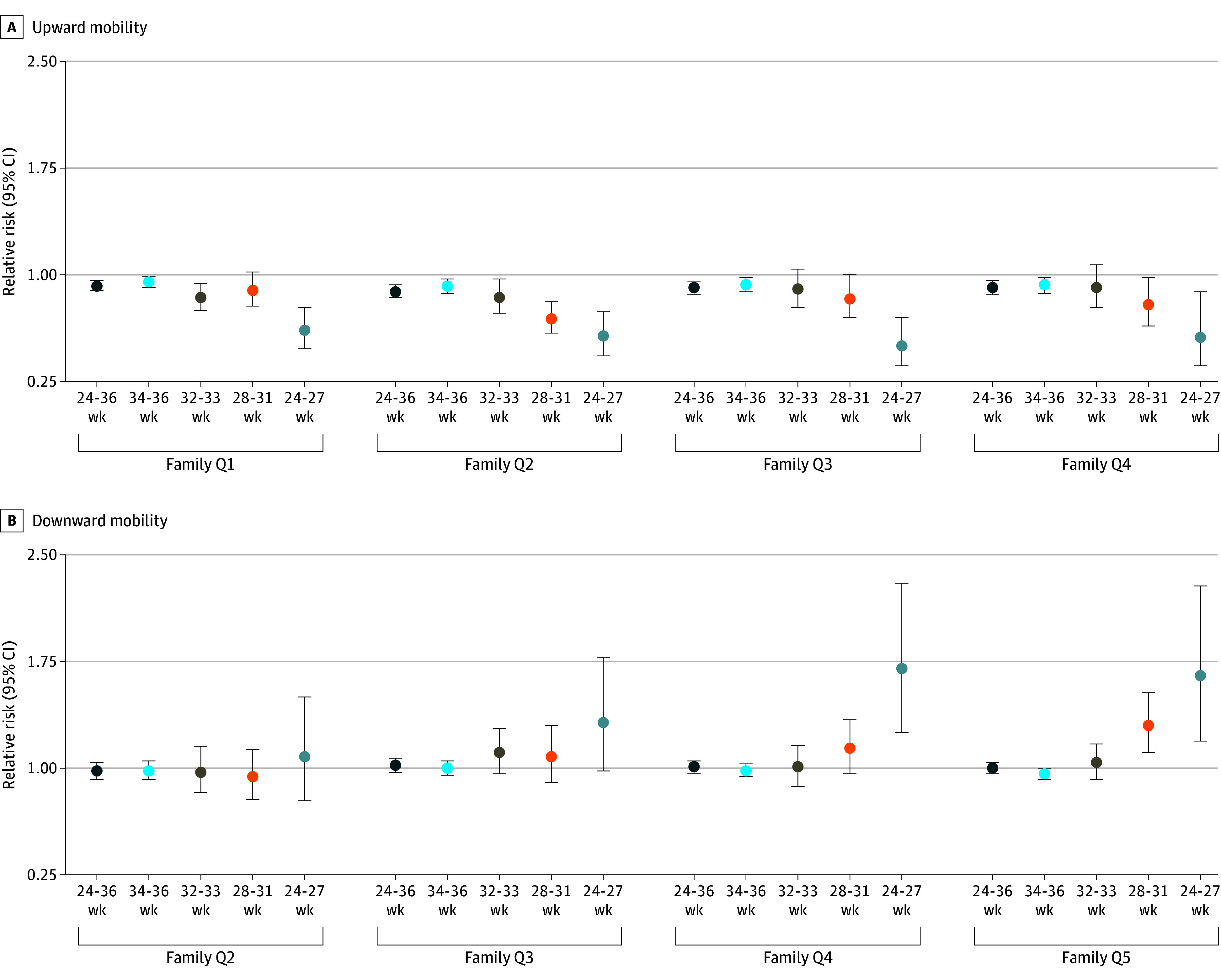
Relative Risk for Associations Between Preterm Birth and Upward or Downward Mobility A, Associations between preterm birth and upward mobility, stratified by family income quintiles at birth, in the matched cohort. B, Associations between preterm birth and downward mobility, stratified by family income quintiles at birth, in the matched cohort. Individuals were categorized into 3 groups according to whether they moved up (upward mobility), down (downward mobility), or stayed in the same quintile (Q) relative to their parent (reference category). Preterm birth was categorized as a binary variable (24-36 weeks vs 37-41 weeks) and a multicategory variable (34-36, 32-33, 28-31, and 24-27 weeks, relative to 37-41 weeks [reference category]).

Preterm-born individuals, especially those with low GA, were generally less likely to transition to a higher SES quintile and more likely to transition to a lower quintile than their parents (Figure 2 and eTable 4 in Supplement 1). For example, the relative risk (RR) for the association between PTB and upward mobility among children of families in the lowest income quintile was 0.92 (95% CI, 0.89-0.96); these differences increased with lower GA (24-27 weeks’ GA: RR, 0.61 [95% CI, 0.48-0.77]). For downward mobility, RRs were close to 1 for PTB and for those born at 34 to 36 weeks’ and 32 to 33 weeks’ GA. However, those born at 28 to 31 weeks’ or 24 to 27 weeks’ GA in families with income in quintiles 4 and 5 had a higher risk of downward mobility, compared with full term–born individuals (24-27 weeks’ GA: RR, 1.65 [95% CI, 1.19-2.28]).

### Secondary Analyses

Analyses using child family income showed similar patterns as the main results (eTables 6 and 7 in Supplement 1). In analyses in which deaths were assigned zero income, differences by PTB were slightly larger, with more pronounced differences in the extremely preterm group (eTables 8 and 9 in Supplement 1). Analyses accounting for missing data using inverse probability of censoring weighting were similar to main results (mean income differences in the matched cohort, −$708.9 [95% CI, −$812 to −$606]; ratio of income per year in the matched cohort, 0.96 [95% CI, 0.96-0.97]), as were analyses restricted to singletons (eTables 10 and 11 in Supplement 1). For sex-stratified analyses, no major differences were noted between females and males (eTables 12 and 13 in Supplement 1). For percentile rank change as the outcome, analyses stratified by age showed slightly larger differences in the group aged 26 to 28 years; analyses stratified by family income quintiles showed smaller differences among children in quintile 5 (eTables 5, 14, and 15 in Supplement 1). Results using income *z* score change as the measure of intergenerational mobility were in the same direction as those using percentile rank change (eTable 16 in Supplement 1).

## Discussion

To our knowledge, this is the first study to use multigenerational linked data to examine the association of PTB with the socioeconomic position of individuals relative to their parents and the role of family SES as a modifying factor. This population-based study showed that preterm-born individuals had lower annual income during young adulthood than those born at full term, with greater differences for those with lower GA. The magnitude of these differences was slightly larger for those belonging to families with low SES. Furthermore, PTB was associated with lower upward mobility and higher downward mobility, particularly for those born at less than 32 weeks’ GA.

Some studies have examined cognitive and educational outcomes during childhood by family SES and showed stronger associations of PTB with adverse cognitive and educational outcomes among children of families with low SES.^19,20^ Furthermore, a systematic review that focused only on individuals born preterm also found lower cognitive scores among preterm-born children born in socioeconomically disadvantaged families.^52^ The present study is the first, to our knowledge, to show that family SES modified the association between PTB and low income in adulthood.

In the context of decreasing upward mobility, it is important to identify early life determinants of social mobility. Evidence suggests that social mobility is unequally distributed across income groups.^53^ For example, Canadian children of parents in lower income groups have lower chances of exceeding the socioeconomic position of their parents.^24^ To our knowledge, only 1 study has assessed the association between PTB and intergenerational mobility.^14^ Using occupation, educational level, and income, all classified into 3 categories, a Finnish study reported that those born late preterm (34-36 weeks’ GA) were less likely to experience upward mobility and more likely to encounter downward mobility, relative to their fathers’ SES status. These findings were generally in the same direction as ours, although our associations for the 34 to 36 weeks’ GA category were smaller in magnitude.

Several lifelong sequelae of PTB might be associated with the lack of upward mobility and the higher risk of downward income mobility observed in this study. Preterm birth may cause numerous adverse health consequences in the child, including cognitive, neurodevelopmental, emotional, and educational challenges.^4,6,7,8,54^ These health and social effects could have a disproportionate association with the socioeconomic position of preterm-born individuals. In addition to the association with children’s SES, PTB might also harm the family’s SES, owing to large health care expenditures and altered working patterns due to the need to dedicate more time and resources to accommodate caregiving and medical needs.^55,56^ These economic challenges might influence parents’ residential choice, subsequently affecting school quality. These challenges might also leave the family with fewer resources available for investments that could enhance intergenerational mobility, such as education, training, and entrepreneurship. Preterm birth might also have a negative association with other aspects of family life, such as breastfeeding, parent-child interaction, parental stress, life satisfaction, and divorce rates, ultimately contributing to adverse developmental and socioeconomic outcomes.^57,58^

By expanding on the existing knowledge of long-term outcomes after PTB, these results could inform policies and interventions to optimize socioeconomic outcomes of preterm-born individuals over their life course, with a special focus on children of families with low SES. Providing parents of preterm-born children with information, resources, and support networks would help them navigate the challenges of caregiving and advocate for their child’s needs. Furthermore, social programs that provide financial assistance and other support to families with preterm-born children can alleviate economic stress, allowing families to invest in education, skills, and other income-improving activities. Policies that promote upward social mobility could help preterm-born individuals overcome the economic challenges they face (eg, by providing them with skill-building opportunities and vocational training to enhance their employability and income prospects).

### Strengths and Limitations

This study has some strengths, including the use of tax data to capture both parental and child income, which reduces self-reporting bias. The study also used population-level data linkages to help achieve a longer duration of follow-up.

Several limitations, however, should be considered when interpreting these results. First, we excluded more than 30% of eligible births for administrative reasons, as these births were not linked to maternal taxes at baseline. We believe this exclusion would not introduce major bias, given that linkage with maternal tax records was mostly associated with birth year (lower linkage rate before 1993). Preterm birth rates in the excluded births were also comparable with those in our sample. We also excluded a small proportion (approximately 3%) due to missing all tax records during the follow-up. These individuals showed sociodemographic differences compared with our sample (more likely to have parents who were young, born outside Canada, and with low SES) and had higher rates of PTB. Possible reasons for missing all tax records include underreporting of early neonatal deaths, which is more likely to occur after PTB, and emigration outside Canada. Although we used matching to account for baseline differences in demographic factors and family income, we cannot rule out residual confounding by other factors, such as parental educational level, occupation, and physical and mental health. Future sibling comparison studies using the same dataset could aid in addressing confounding by shared family characteristics.

We used only income as a measure of socioeconomic position, but income may not fully capture all aspects of SES or intergenerational mobility. Measurement errors in GA are likely because we obtained GA from birth records that relied on physician-reported or parent-reported data. Measurement errors for family income are also possible because we used income reported on tax files at baseline as a proxy for the family’s permanent income. We, however, averaged parental income over 2 years to reduce the risk of bias. For children, we considered income over only a limited part of the life cycle, making the results vulnerable to life-cycle bias. Previous studies have suggested that life-cycle bias is minimized when children are in their late 30s and early 40s.^23,59^ However, using income rank instead of actual income would reduce this bias, as empirical evidence suggests that income ranks are established at an early stage of life and remain relatively stable afterward.^23^ Furthermore, results stratified by age and those using income *z* scores were generally in the same direction.

## Conclusions

This population-based intergenerational cohort study suggests that PTB was associated with lower income during young adulthood, especially with lower GA, with more differences for children of families near the bottom of income distribution. Furthermore, preterm-born individuals, particularly those born very or extremely preterm in economically disadvantaged families, had less potential for social mobility. Future studies should consider longer durations of follow-up to examine intergenerational income mobility when children are in their 30s and 40s. Studies should also consider other aspects of SES, such as education and occupation.

## Notes

Supplement 1. **eTable 1.** Description of Different Datasets at Statistics Canada Used in the Study and the Relevant Study Variables **eTable 2.** Characteristics of Individuals Included vs Excluded From the Study Population [N (%)] **eTable 3.** Characteristics of the Unmatched (a) and Matched Cohorts (b) According to Gestational Age Category [N (%)] **eTable 4.** The Transition Matrix of Probabilities That the Individual's Income Observed in a Specific Quintile at the End of Follow-Up, Conditional on Their Parents’ Quintile at Baseline in the Matched Cohorts by PTB (and GA Subcategories) **eTable 5.** Coefficients and Standard Errors for the Association Between Gestational Age Categories and Annual Income in the Matched Cohort*,* in Analyses Including Cross Product Terms Between Gestational Age Categories (GA) and Family Income Quintiles **eTable 6.** Associations Between Preterm Birth and Annual Family Income at or After the Age of 18 Years for Individuals Born in 1990-1996 in Canada, Overall and Stratified by Family Income Quintiles at Baseline **eTable 7**. Associations Between Preterm Birth and Mean Difference in Percentile Rank Change (Calculated Using Family Income) for Individuals Born in 1990-1996 in Canada **eTable 8.** Associations Between Preterm Birth and Annual Income at or After the Age of 18 Years When Individuals Who Died Were Assigned Zero Income, Overall and Stratified by Family Income Quintiles at Baseline **eTable 9.** Associations Between Preterm Birth and Mean Difference in Percentile Rank Change When Individuals Who Died Were Assigned Lowest Percentile **eTable 10.** Associations Between Preterm Birth and Annual Income at or After the Age of 18 Years, Overall and Stratified by Family Income Quintiles at Baseline, Only Among Singletons **eTable 11.** Associations Between Preterm Birth and Mean Difference in Percentile Rank Change Only Among Singletons **eTable 12.** Associations Between Preterm Birth and Annual Income at or After the Age of 18 Years for Individuals Born in 1990-1996 in Canada, Stratified by Sex **eTable 13.** Associations Between Preterm Birth and Mean Difference in Percentile Rank Change for Individuals Born in 1990-1996 in Canada in the Matched Cohort, Stratified by Sex **eTable 14.** Associations Between Preterm Birth and Mean Difference in Percentile Rank Change for Individuals Born in 1990-1996 in Canada in the Matched Cohort, Stratified by Family Income Quintile at Baseline **eTable 15.** Associations Between Preterm Birth and Mean Difference in Percentile Rank Change for Individuals Born in 1990-1996 in Canada in the Matched Cohort, Stratified by Age Group **eTable 16.** Associations Between Preterm Birth and Mean Difference in Income-z-Score Change for Individuals Born in 1990-1996 in Canada in the Matched Cohort

Supplement 2.
Data Sharing Statement
