# Supplementary material for: Preterm Birth, Family Income, and Intergenerational Income Mobility
Source: JAMA Netw Open. 2024 Jun 10;7(6):e2415921. doi: 10.1001/jamanetworkopen.2024.15921 (PMC11165381; doi:10.1001/jamanetworkopen.2024.15921)
Supplement: Supplement 1. — eTable 1. Description of Different Datasets at Statistics Canada Used in the Study and the Relevant Study Variables eTable 2. Characteristics of Individuals Included vs Excluded From the Study Population [N (%)] eTable 3. Characteristics of the Unmatched (a) and Matched Cohorts (b) According to Gestational Age Category [N (%)] eTable 4. The Transition Matrix of Probabilities That the Individual's Income Observed in a Specific Quintile at the End of Follow-Up, Conditional on Their Parents’ Quintile at Baseline in the Matched Cohorts by PTB (and GA Subcategories) eTable 5. Coefficients and Standard Errors for the Association Between Gestational Age Categories and Annual Income in the Matched Cohort, in Analyses Including Cross Product Terms Between Gestational Age Categories (GA) and Family Income Quintiles eTable 6. Associations Between Preterm Birth and Annual Family Income at or After the Age of 18 Years for Individuals Born in 1990-1996 in Canada, Overall and Stratified by Family Income Quintiles at Baseline eTable 7. Associations Between Preterm Birth and Mean Difference in Percentile Rank Change (Calculated Using Family Income) for Individuals Born in 1990-1996 in Canada eTable 8. Associations Between Preterm Birth and Annual Income at or After the Age of 18 Years When Individuals Who Died Were Assigned Zero Income, Overall and Stratified by Family Income Quintiles at Baseline eTable 9. Associations Between Preterm Birth and Mean Difference in Percentile Rank Change When Individuals Who Died Were Assigned Lowest Percentile eTable 10. Associations Between Preterm Birth and Annual Income at or After the Age of 18 Years, Overall and Stratified by Family Income Quintiles at Baseline, Only Among Singletons eTable 11. Associations Between Preterm Birth and Mean Difference in Percentile Rank Change Only Among Singletons eTable 12. Associations Between Preterm Birth and Annual Income at or After the Age of 18 Years for Individuals Born in 1990-1996 in Canada, Stratified [file jamanetwopen-e2415921-s001.pdf]

Supplementary Online Content

Ahmed A, Pullenayegum E, McDonald SD, et al. Preterm birth, family income, and intergenerational income mobility. *JAMA Netw Open.* 2024;7(6):e2415921. doi:10.1001/jamanetworkopen.2024.15921

eTable 1. Description of Different Datasets at Statistics Canada Used in the Study and the Relevant Study Variables
eTable 2. Characteristics of Individuals Included vs Excluded From the Study Population [N (%)]
eTable 3. Characteristics of the Unmatched (a) and Matched Cohorts (b) According to Gestational Age Category [N (%)]
eTable 4. The Transition Matrix of Probabilities That the Individual's Income Observed in a Specific Quintile at the End of Follow-Up, Conditional on Their Parents' Quintile at Baseline in the Matched Cohorts by PTB (and GA Subcategories)
eTable 5. Coefficients and Standard Errors for the Association Between Gestational Age Categories and Annual Income in the Matched Cohort, in Analyses Including Cross Product Terms Between Gestational Age Categories (GA) and Family Income Quintiles
eTable 6. Associations Between Preterm Birth and Annual Family Income at or After the Age of 18 Years for Individuals Born in 1990-1996 in Canada, Overall and Stratified by Family Income Quintiles at Baseline
eTable 7. Associations Between Preterm Birth and Mean Difference in Percentile Rank Change (Calculated Using Family Income) for Individuals Born in 1990-1996 in Canada
eTable 8. Associations Between Preterm Birth and Annual Income at or After the Age of 18 Years When Individuals Who Died Were Assigned Zero Income, Overall and Stratified by Family Income Quintiles at Baseline
eTable 9. Associations Between Preterm Birth and Mean Difference in Percentile Rank Change When Individuals Who Died Were Assigned Lowest Percentile
eTable 10. Associations Between Preterm Birth and Annual Income at or After the Age of 18 Years, Overall and Stratified by Family Income Quintiles at Baseline, Only Among Singletons
eTable 11. Associations Between Preterm Birth and Mean Difference in Percentile Rank Change Only Among Singletons
eTable 12. Associations Between Preterm Birth and Annual Income at or After the Age of 18 Years for Individuals Born in 1990-1996 in Canada, Stratified by Sex
eTable 13. Associations Between Preterm Birth and Mean Difference in Percentile Rank Change for Individuals Born in 1990-1996 in Canada in the Matched Cohort, Stratified by Sex
eTable 14. Associations Between Preterm Birth and Mean Difference in Percentile Rank Change for Individuals Born in 1990-1996 in Canada in the Matched Cohort, Stratified by Family Income Quintile at Baseline
eTable 15. Associations Between Preterm Birth and Mean Difference in Percentile Rank Change for Individuals Born in 1990-1996 in Canada in the Matched Cohort, Stratified by Age Group
eTable 16. Associations Between Preterm Birth and Mean Difference in Income-z-Score Change for Individuals Born in 1990-1996 in Canada in the Matched Cohort

This supplementary material has been provided by the authors to give readers additional information about their work.

© 2024 Ahmed A et al. *JAMA Network Open.*

eTable 1. Description of Different Datasets at Statistics Canada Used in the Study and the Relevant Study Variables

**Name of the dataset**	**Brief description**	**Study variables**
**The Vital Statistics-Birth (VSB) file**	A cross-sectional administrative database that collects data on all live births in Canada from all provincial and territorial vital statistics registries.	Exposure: gestational age.<br>Matching variables: individual's sex (female or male), the birth plurality (single or multiple), province of birth, and birth year, mother's marital status (single, married, other [includes widowed, divorced, separated], or missing), parental age (<20, 20-24, 25-29, 30-34, 35-39, >40 years for the mother and <25, 25–29,30–34, 35–39, >40 years, or missing for the father), parental place of birth based on Statistical Classification of Countries and Areas of Interest for Social Statistics (Canada, North America excluding Canada, Central and South America, Europe, Africa, Asia, and other [Oceania/Antarctica and adjacent islands, missing, or unknown]), and maternal parity (0, 1, 2, 3, or ≥4 previous live births).
**The Vital Statistics- Death (VSD) file**	A cross-sectional, administrative database that collects mortality data from all provincial and territorial vital statistics registries.	Mortality during the time of follow-up.
**The TI Family File (TIFF)**	The T1FF includes data on all Canadian residents who filed their tax return (T1 form) or received the Canada Child Tax Benefit in a given year with their spouses and children. The T1FF contains demographic information and data on income and household composition.	Outcomes: Annual personal and family income; family income at baseline.<br>Matching variables: family income quintiles at baseline, maternal place of residence (rural/urban).

© 2024 Ahmed A et al. *JAMA Network Open.*

eTable 2. Characteristics of Individuals Included vs Excluded From the Study Population [N (%)]

Characteristics	Included sample (n= 1,625,480)	Excluded due to no linkage to maternal tax records at baseline (n=878,790)	SMD	Excluded due to missing tax records (n= 76,730)	SMD
Individual's sex			0.00		0.08
Female	794,540 (48.9)	427,580 (48.7)		34,410 (44.8)	
Male	830,940 (51.1)	451,200 (51.3)		42,330 (55.2)	
Maternal parity			0.26		0.12
0	767,500 (47.2)	307,780 (35.0)		34,610 (45.1)	
1	545,400 (33.6)	344,910 (39.2)		24,520 (32.0)	
2	212,300 (13.1)	156,550 (17.8)		10,660 (13.9)	
3	65,400 (4.0)	47,700 (5.4)		3,970 (5.2)	
≥4	34,870 (2.1)	21,860 (2.5)		2,980 (3.9)	
Maternal age			0.12		0.12
<20 years	85,990 (5.3)	64,540 (7.3)		5,690 (7.4)	
20-24 years	323,970 (19.9)	158,320 (18)		16,730 (21.8)	
25-29 years	588,430 (36.2)	292,480 (33.3)		25,250 (32.9)	
30-34 years	462,150 (28.4)	255,850 (29.1)		20,790 (27.1)	
35-39 years	145,960 (9.0)	93,690 (10.7)		7,210 (9.4)	
≥40 years	18,980 (1.2)	13,900 (1.6)		1,070 (1.4)	
Paternal age			0.18		0.28
<25 years	183,560 (11.3)	90,660 (10.3)		8,580 (11.2)	
25–29 years	468,550 (28.8)	215,480 (24.5)		18,540 (24.2)	
30–34 years	531,100 (32.7)	274,610 (31.2)		22,470 (29.3)	
35–39 years	247,810 (15.2)	145,670 (16.6)		11,480 (15.0)	
≥40 years	100,210 (6.2)	67,700 (7.7)		5,280 (6.9)	
Missing	94,250 (5.8)	84,680 (9.6)		10,400 (13.6)	
Place of birth			0.18		0.23
Alberta	181,840 (11.2)	87,060 (9.9)		7,810 (10.2)	
Atlantic Provinces	120,880 (7.4)	61,330 (7.0)		3,320 (4.3)	
British Columbia	200,990 (12.4)	97,020 (11.0)		10,290 (13.4)	
Manitoba	75,210 (4.6)	33,280 (3.8)		3,030 (3.9)	
Ontario	574,380 (35.3)	384,630 (43.8)		24,890 (32.4)	
Quebec	400,080 (24.6)	181,160 (20.6)		24,960 (32.5)	
Saskatchewan	65,540 (4.0)	29,190 (3.3)		1,990 (2.6)	
Yukon, Nunavut, and Northwest Territory	6,580 (0.4)	5,130 (0.6)		440 (0.6)	
Marital status			0.11		0.17
married	1,104,640 (68.0)	606,520 (69.0)		46,510 (60.6)	

© 2024 Ahmed A et al. *JAMA Network Open.*

missing	91,160 (5.6)	29,700 (3.4)		6,740 (8.8)	
Other	29,880 (1.8)	17,120 (1.9)		1,800 (2.3)	
Single	399,810 (24.6)	225,460 (25.7)		21,680 (28.3)	
Birth year			1.63		0.14
1990	97,450 (6.0)	271,950 (30.9)		3,550 (4.6)	
1991	127,180 (7.8)	243,420 (27.7)		4,940 (6.4)	
1992	139,210 (8.6)	233,120 (26.5)		5,330 (6.9)	
1993	315,410 (19.4)	36,800 (4.2)		14,690 (19.1)	
1994	322,090 (19.8)	32,410 (3.7)		14,830 (19.3)	
1995	316,810 (19.5)	32,090 (3.7)		15,770 (20.6)	
1996	307,330 (18.9)	29,000 (3.3)		17,620 (23.0)	
Maternal place of birth			0.16		0.49
Africa	11,600 (0.7)	6,140 (0.7)		1,910 (2.5)	
Asia	91,490 (5.6)	39,600 (4.5)		8,470 (11.0)	
Canada	1,319,980 (81.2)	680,580 (77.4)		46,270 (60.3)	
Central and South America	26,570 (1.6)	11,630 (1.3)		3,390 (4.4)	
Europe	73,810 (4.5)	53,640 (6.1)		5,420 (7.1)	
North America	18,630 (1.1)	14,970 (1.7)		3,140 (4.1)	
Other	83,410 (5.1)	72,230 (8.2)		8,150 (10.6)	
Paternal place of birth			0.21		0.57
Africa	13,670 (0.8)	6,930 (0.8)		2,080 (2.7)	
Asia	88,440 (5.4)	38,600 (4.4)		8,280 (10.8)	
Canada	1,222,740 (75.2)	602,180 (68.5)		38,030 (49.6)	
Central and South America	27,240 (1.7)	10,650 (1.2)		3,080 (4.0)	
Europe	84,930 (5.2)	59,410 (6.8)		5,540 (7.2)	
North America	15,220 (0.9)	11,710 (1.3)		2,770 (3.6)	
Other	173,240 (10.7)	149,320 (17)		16,950 (22.1)	
Birth plurality			0.05		0.17
Singleton	1,594,780 (98.1)	855,940 (97.4)		72,550 (94.6)	
Multiple	30,700 (1.9)	22,850 (2.6)		4,180 (5.4)	
Maternal place of residence			-		0.19
Urban	1,221,160 (75.1)	-		63,620 (82.9)	
Rural	404,320 (24.9)	-		13,120 (17.1)	
Family income quintile at baseline			-		0.38
Q1 (lowest)	312,730 (19.2)	-		26,680 (34.8)	
Q2	324,950 (20.0)	-		15,810 (20.6)	
Q3	328,680 (20.2)	-		12,190 (15.9)	
Q4	330,490 (20.3)	-		10,460 (13.6)	
Q5 (highest)	328,650 (20.2)	-		11,600 (15.1)	
Preterm birth			0.04		0.11
Preterm 24-36	111,820 (6.9)	68,440 (7.8)		7,640 (10.0)	

© 2024 Ahmed A et al. *JAMA Network Open.*

Term 37-41	2,259,060 (93.1)	810,340 (92.2)		69,090 (90.0)	
Gestational age categories			0.05		0.12
37-41	1,513,670 (93.1)	810,340 (92.2)		69,090 (90.0)	
34-36	87,540 (5.4)	49,720 (5.7)		5,450 (7.1)	
32-33	12,130 (0.7)	8,690 (1.0)		920 (1.2)	
28-31	8,590 (0.5)	6,700 (0.8)		790 (1.0)	
24-27	3,560 (0.2)	3,330 (0.4)		500 (0.6)	

Note: All numbers were rounded to the nearest ten for confidentiality reasons

© 2024 Ahmed A et al. *JAMA Network Open.*

eTable 3. Characteristics of the Unmatched (a) and Matched Cohorts (b) According to Gestational Age Category [N (%)]

(a) Before matching

	**37-41 weeks (reference, n=1,513,670)**	**34-36 weeks w (n= 87,540)**		**32-33 weeks (n= 12,130)**		**28-31 weeks (n= 8,590)**		**24-27 weeks (n= 3,560)**	
**Characteristics**	**N (%)**	**N (%)**	**SMD**	**N (%)**	**SDM**	**N (%)**	**SDM**	**N (%)**	**SDM**
Individual's sex			0.06		0.07		0.08		0.04
Female	743,140 (49.1)	40330 (46.1)		5510 (45.4)		3890 (45.2)		1680 (47.2)	
Male	770,530 (50.9)	47210 (53.9)		6630 (54.6)		4700 (54.8)		1880 (52.8)	
Birth plurality			0.43		0.65		0.70		0.65
Singleton	1,497,750 (98.9)	77800 (88.9)		9720 (80.1)		6670 (77.7)		2850 (80.0)	
Multiple	15,920 (1.1)	9740 (11.1)		2420 (19.9)		1920 (22.3)		710 (20.0)	
Maternal parity			0.08		0.10		0.11		0.09
0	712,990 (47.1)	42780 (48.9)		5880 (48.4)		4140 (48.2)		1720 (48.4)	
1	511,330 (33.8)	26650 (30.4)		3660 (30.2)		2640 (30.7)		1130 (31.8)	
2	197,460 (13)	11640 (13.3)		1670 (13.7)		1120 (13.0)		430 (11.9)	
3	60,190 (4.0)	4080 (4.7)		570 (4.7)		390 (4.6)		170 (4.9)	
≥4	31,710 (2.1)	2400 (2.7)		360 (3.0)		300 (3.5)		110 (3.0)	
Maternal age			0.09		0.13		0.17		0.17
<20 years	79,080 (5.2)	5240 (6.0)		770 (6.3)		640 (7.5)		260 (7.4)	
20-24 years	301,690 (19.9)	17460 (19.9)		2410 (19.9)		1680 (19.5)		730 (20.5)	
25-29 years	551,210 (36.4)	29560 (33.8)		3910 (32.2)		2680 (31.2)		1090 (30.6)	
30-34 years	430,810 (28.5)	24460 (27.9)		3440 (28.4)		2440 (28.4)		1010 (28.3)	
35-39 years	133,830 (8.8)	9340 (10.7)		1380 (11.4)		990 (11.5)		420 (11.9)	
≥40 years	17,060 (1.1)	1480 (1.7)		230 (1.9)		170 (1.9)		50 (1.3)	
Paternal age			0.09		0.14		0.16		0.16
<25 years	170,260 (11.2)	10240 (11.7)		1490 (12.2)		1110 (12.9)		480 (13.4)	
25–29 years	438,380 (29.0)	23900 (27.3)		3170 (26.1)		2190 (25.5)		910 (25.6)	
30–34 years	496,670 (32.8)	27170 (31.0)		3670 (30.3)		2540 (29.6)		1050 (29.5)	
35–39 years	230,290 (15.2)	13720 (15.7)		1890 (15.6)		1350 (15.8)		560 (15.6)	
≥40 years	92,060 (6.1)	6320 (7.2)		920 (7.6)		660 (7.7)		250 (7.1)	
Missing	86,020 (5.7)	6200 (7.1)		1000 (8.2)		730 (8.5)		310 (8.8)	
Maternal place of birth			0.19		0.10		0.08		0.13
Africa	10,620 (0.7)	760 (0.9)		90 (0.8)		80 (0.9)		50 (1.3)	
Asia	83,150 (5.5)	6840 (7.8)		820 (6.7)		500 (5.8)		180 (5.2)	
Canada	1,234,900 (81.6)	65790 (75.1)		9600 (79.1)		6880 (80.1)		2820 (79.1)	
Central and South America	24,010 (1.6)	1930 (2.2)		300 (2.5)		210 (2.4)		120 (3.3)	
Europe	68,880 (4.6)	3920 (4.5)		490 (4.1)		360 (4.2)		160 (4.4)	
North America	17,550 (1.2)	850 (1.0)		110 (0.9)		90 (1.0)		50 (1.3)	
Other	74,560 (4.9)	7460 (8.5)		720 (5.9)		480 (5.6)		190 (5.5)	

© 2024 Ahmed A et al. *JAMA Network Open.*

Paternal place of birth			0.18		0.13		0.13		0.17
Africa	12,620 (0.8)	820 (0.9)		100 (0.8)		90 (1.0)		40 (1.2)	
Asia	80,590 (5.3)	6400 (7.3)		790 (6.5)		470 (5.4)		190 (5.4)	
Canada	1,145,150 (75.7)	60190 (68.8)		8670 (71.5)		6200 (72.2)		2530 (71.2)	
Central and South America	24,750 (1.6)	1870 (2.1)		300 (2.5)		220 (2.6)		110 (3.0)	
Europe	79,420 (5.2)	4430 (5.1)		550 (4.6)		390 (4.6)		140 (3.9)	
North America	14,300 (0.9)	710 (0.8)		120 (1.0)		60 (0.7)		30 (1.0)	
Other	156,840 (10.4)	13120 (15.0)		1600 (13.2)		1170 (13.6)		510 (14.4)	
Maternal marital status at birth			0.12		0.21		0.24		0.26
Married	1,034,400 (68.3)	55870 (63.8)		7260 (59.8)		5050 (58.8)		2070 (58.1)	
Other	81,900 (5.4)	6750 (7.7)		1190 (9.8)		910 (10.6)		410 (11.5)	
Missing	27,470 (1.8)	1850 (2.1)		260 (2.2)		200 (2.4)		90 (2.5)	
Single	369,900 (24.4)	23070 (26.4)		3420 (28.2)		2430 (28.3)		990 (27.8)	
Place of birth			0.12		0.09		0.13		0.20
Alberta	169,490 (11.2)	9150 (10.4)		1530 (12.6)		1160 (13.5)		520 (14.6)	
Atlantic Provinces	112,730 (7.4)	6150 (7.0)		970 (8.0)		740 (8.6)		299 (8.4)[a]	
British Columbia	188,710 (12.5)	9190 (10.5)		1540 (12.7)		1060 (12.4)		480 (13.6)	
Manitoba	69,820 (4.6)	4050 (4.6)		630 (5.2)		500 (5.8)		220 (6.1)	
Ontario	530,330 (35.0)	35660 (40.7)		4310 (35.5)		2920 (34)		1170 (32.8)	
Quebec	375,020 (24.8)	19930 (22.8)		2640 (21.7)		1810 (21.0)		690 (19.3)	
Saskatchewan	61,340 (4.1)	3140 (3.6)		490 (4.1)		380 (4.5)		190 (5.2)	
Territories	6,230 (0.4)	290 (0.3)		30 (0.2)		20 (0.2)		299 (8.4)[a]	
Birth year			0.12		0.21		0.21		0.20
1990	92,550 (6.1)	4010 (4.6)		440 (3.7)		310 (3.6)		140 (4.0)	
1991	120,180 (7.9)	5680 (6.5)		660 (5.4)		470 (5.5)		190 (5.4)	
1992	131,420 (8.7)	6360 (7.3)		700 (5.7)		510 (5.9)		230 (6.4)	
1993	293,840 (19.4)	16880 (19.3)		2400 (19.8)		1660 (19.3)		640 (18.0)	
1994	299,070 (19.8)	17690 (20.2)		2660 (21.9)		1900 (22.1)		770 (21.7)	
1995	292,950 (19.4)	18550 (21.2)		2670 (22.0)		1850 (21.6)		790 (22.2)	
1996	283,660 (18.7)	18380 (21.0)		2600 (21.4)		1890 (22.0)		790 (22.3)	
Maternal place of residence			0.06		0.03		0.03		0.04
Urban	1,134,640 (75.0)	68000 (77.7)		9240 (76.2)		6560 (76.4)		2720 (76.5)	
Rural	379,020 (25.0)	19550 (22.3)		2890 (23.8)		2030 (23.6)		840 (23.5)	
Family income quintile at baseline			0.12		0.12		0.14		0.15
Q1 (lowest)	286,850 (19.0)	20190 (23.1)		2810 (23.2)		2050 (23.9)		830 (23.2)	
Q2	301,380 (19.9)	18450 (21.1)		2530 (20.8)		1800 (20.9)		800 (22.5)	
Q3	307,230 (20.3)	16950 (19.4)		2260 (18.6)		1600 (18.7)		640 (17.9)	
Q4	309,770 (20.5)	16120 (18.4)		2300 (19.0)		1620 (18.8)		680 (19.2)	
Q5 (highest)	308,450 (20.4)	15840 (18.1)		2230 (18.4)		1520 (17.7)		610 (17.2)	

Note: All numbers were rounded to the nearest ten for confidentiality reasons

[a] Atlantic provinces and Territories were combined due to small cell count

© 2024 Ahmed A et al. *JAMA Network Open.*

(b) After matching [weighted]

	34-36 weeks		32-33 weeks		28-31 weeks		24-27 weeks	
**Characteristics**	**Term (n=832,120)**	**Preterm (n= 70,580)**	**Term (n= 307,520)**	**Preterm (n= 8,990)**	**Term (n= 224,930)**	**Preterm (n= 6,190)**	**Term (n= 101,640)**	**Preterm (n= 2,560)**
Individual's sex								
Female	380610 (45.7)	32280 (45.7)	137310 (44.7)	4020 (44.7)	99980 (44.4)	2750 (44.4)	47360 (46.6)	1190 (46.6)
Male	451510 (54.3)	38300 (54.3)	170210 (55.3)	4980 (55.3)	124950 (55.6)	3440 (55.6)	54280 (53.4)	1370 (53.4)
Birth plurality								
Singleton	801010 (96.3)	67940 (96.3)	285740 (92.9)	8360 (92.9)	207780 (92.4)	5720 (92.4)	94810 (93.3)	2390 (93.3)
Multiple	31110 (3.7)	2640 (3.7)	21780 (7.1)	640 (7.1)	17150 (7.6)	470 (7.6)	6830 (6.7)	170 (6.7)
Maternal parity								
0	438600 (52.7)	37200 (52.7)	165350 (53.8)	4840 (53.8)	121420 (54.0)	3340 (54.0)	54040 (53.2)	1360 (53.2)
1	252350 (30.3)	21410 (30.3)	90300 (29.4)	2640 (29.4)	66150 (29.4)	1820 (29.4)	30830 (30.3)	780 (30.3)
2	98770 (11.9)	8380 (11.9)	36690 (11.9)	1070 (11.9)	25620 (11.4)	710 (11.4)	10890 (10.7)	270 (10.7)
3	27710 (3.3)	2350 (3.3)	9680 (3.1)	280 (3.1)	7520 (3.3)	210 (3.3)	3810 (3.8)	100 (3.8)
≥4	14690 (1.8)	1250 (1.8)	5500 (1.8)	160 (1.8)	4220 (1.9)	120 (1.9)	2070 (2.0)	50 (2.0)
Maternal age								
<20 years	50130 (6.0)	4250 (6.0)	19800 (6.4)	580 (6.4)	17370 (7.7)	480 (7.7)	7510 (7.4)	190 (7.4)
20-24 years	168130 (20.2)	14260 (20.2)	62130 (20.2)	1820 (20.2)	44080 (19.6)	1210 (19.6)	20900 (20.6)	530 (20.6)
25-29 years	294510 (35.4)	24980 (35.4)	103570 (33.7)	3030 (33.7)	73380 (32.6)	2020 (32.6)	32260 (31.7)	810 (31.7)
30-34 years	235620 (28.3)	19990 (28.3)	88730 (28.9)	2600 (28.9)	64980 (28.9)	1790 (28.9)	29800 (29.3)	750 (29.3)
35-39 years	76550 (9.2)	6490 (9.2)	29990 (9.8)	880 (9.8)	22280 (9.9)	610 (9.9)	10450 (10.3)	280 (11.0)[a]
≥40 years	7180 (0.9)	610 (0.9)	3320 (1.1)	100 (1.1)	2830 (1.3)	80 (1.3)	720 (0.7)	280 (11.0)[a]
Paternal age								
<25 years	97750 (11.7)	8290 (11.7)	39120 (12.7)	1140 (12.7)	29840 (13.3)	820 (13.3)	12950 (12.7)	330 (12.7)
25–29 years	237640 (28.6)	20160 (28.6)	83630 (27.2)	2450 (27.2)	59200 (26.3)	1630 (26.3)	27340 (26.9)	690 (26.9)
30–34 years	269350 (32.4)	22850 (32.4)	97480 (31.7)	2850 (31.7)	70180 (31.2)	1930 (31.2)	31590 (31.1)	800 (31.1)
35–39 years	123990 (14.9)	10520 (14.9)	45510 (14.8)	1330 (14.8)	34160 (15.2)	940 (15.2)	15100 (14.9)	380 (14.9)
≥40 years	46630 (5.6)	3960 (5.6)	18090 (5.9)	530 (5.9)	12790 (5.7)	350 (5.7)	6080 (6.0)	150 (6.0)
Missing	56770 (6.8)	4820 (6.8)	23690 (7.7)	690 (7.7)	18750 (8.3)	520 (8.3)	8580 (8.4)	220 (8.4)
Maternal place of birth								
Africa	3770 (0.5)	320 (0.5)	890 (0.3)	30 (0.3)	1050 (0.5)	30 (0.5)	640 (0.6)	30 (1.0)[c]
Asia	60630 (7.3)	5140 (7.3)	19350 (6.3)	570 (6.3)	11010 (4.9)	300 (4.9)	5130 (5.0)	130 (5.0)
Canada	655230 (78.7)	55580 (78.7)	256270 (83.3)	7500 (83.3)	190700 (84.8)	5250 (84.8)	85070 (83.7)	2140 (83.7)
Central and	12060 (1.4)	1020 (1.4)	4990 (1.6)	150 (1.6)	4110 (1.8)	110 (1.8)	2540 (2.5)	60 (2.5)
South America	27150 (3.3)	2300 (3.3)	7970 (2.6)	230 (2.6)	5340 (2.4)	150 (2.4)	2380 (2.3)	60 (2.3)
Europe	2900 (0.3)	250 (0.3)	680 (0.2)	530 (5.8)[b]	440 (0.2)	350 (5.7)[b]	440 (0.4)	30 (1.0)[c]
North America	70370 (8.5)	5970 (8.5)	17370 (5.6)	530 (5.8)[b]	12280 (5.5)	350 (5.7)[b]	5440 (5.4)	140 (5.4)

© 2024 Ahmed A et al. *JAMA Network Open.*

Other								
Paternal place of birth								
Africa	4130 (0.5)	350 (0.5)	920 (0.3)	30 (0.3)	1160 (0.5)	30 (0.5)	760 (0.7)	30 (0.9)[c]
Asia	58090 (7.0)	4930 (7.0)	18940 (6.2)	550 (6.2)	10610 (4.7)	290 (4.7)	4970 (4.9)	130 (4.9)
Canada	601950 (72.3)	51060 (72.3)	232740 (75.7)	6810 (75.7)	172120 (76.5)	4740 (76.5)	76650 (75.4)	1930 (75.4)
Central and	11620 (1.4)	990 (1.4)	5090 (1.7)	150 (1.7)	4180 (1.9)	120 (1.9)	2380 (2.3)	60 (2.3)
South America	31470 (3.8)	2670 (3.8)	9640 (3.1)	280 (3.1)	6610 (2.9)	180 (2.9)	2940 (2.9)	70 (2.9)
Europe	2290 (0.3)	190 (0.3)	620 (0.2)	1180 (13.1)[b]	620 (0.3)	840 (13.5)[b]	240 (0.2)	30 (0.9)[c]
North America	122570 (14.7)	10400 (14.7)	39560 (12.9)	1180 (13.1)[b]	29620 (13.2)	840 (13.5)[b]	13710 (13.5)	350 (13.5)
Other								
Marital status								
Married	545390 (65.5)	46260 (65.5)	191170 (62.2)	5590 (62.2)	137850 (61.3)	3790 (61.3)	60910 (59.9)	1530 (59.9)
Other	55400 (6.7)	4700 (6.7)	25340 (8.2)	740 (8.2)	20320 (9)	560 (9)	9930 (9.8)	250 (9.8)
Missing	8710 (1.0)	740 (1.0)	3320 (1.1)	100 (1.1)	2540 (1.1)	70 (1.1)	990 (1.0)	30 (1.0)
Single	222620 (26.8)	18880 (26.8)	87700 (28.5)	2570 (28.5)	64220 (28.6)	1770 (28.6)	29800 (29.3)	750 (29.3)
Place of birth								
Alberta	81770 (9.8)	6940 (9.8)	37510 (12.2)	1100 (12.2)	29770 (13.2)	820 (13.2)	15100 (14.9)	380 (14.9)
Atlantic	57840 (7.0)	4910 (7.0)	24720 (8.0)	730 (8.1)[d]	19260 (8.6)	540 (8.7)[d]	8660 (8.5)[d]	220 (8.5)[d]
Provinces	78510 (9.4)	6660 (9.4)	34260 (11.1)	1000 (11.1)	25400 (11.3)	700 (11.3)	13350 (13.1)	340 (13.1)
British	33780 (4.1)	2870 (4.1)	13880 (4.5)	410 (4.5)	11050 (4.9)	300 (4.9)	6040 (5.9)	150 (5.9)
Columbia	353260 (42.5)	29960 (42.5)	113890 (37)	3330 (37)	78970 (35.1)	2170 (35.1)	33730 (33.2)	850 (33.2)
Manitoba	198620 (23.9)	16850 (23.9)	71320 (23.2)	2090 (23.2)	50480 (22.4)	1390 (22.4)	20260 (19.9)	510 (19.9)
Ontario	26970 (3.2)	2290 (3.2)	11590 (3.8)	340 (3.8)	9740 (4.3)	270 (4.3)	4490 (4.4)	110 (4.4)
Quebec	1380 (0.2)	120 (0.2)	340 (0.1)	730 (8.1)[d]	250 (0.1)	540 (8.7)[d]	8660 (8.5)[d]	220 (8.5)[d]
Saskatchewan								
Territories								
Birth year								
1990	37950 (4.6)	3220 (4.6)	10330 (3.4)	300 (3.4)	8140 (3.6)	220 (3.6)	4050 (4.0)	100 (4.0)
1991	54080 (6.5)	4590 (6.5)	17130 (5.6)	500 (5.6)	12570 (5.6)	350 (5.6)	5400 (5.3)	140 (5.3)
1992	61490 (7.4)	5220 (7.4)	17850 (5.8)	520 (5.8)	13770 (6.1)	380 (6.1)	5880 (5.8)	150 (5.8)
1993	161070 (19.4)	13660 (19.4)	61960 (20.1)	1810 (20.1)	44270 (19.7)	1220 (19.7)	18720 (18.4)	470 (18.4)
1994	168220 (20.2)	14270 (20.2)	68180 (22.2)	1990 (22.2)	49830 (22.2)	1370 (22.2)	22810 (22.4)	570 (22.4)
1995	176380 (21.2)	14960 (21.2)	66260 (21.5)	1940 (21.5)	49750 (22.1)	1370 (22.1)	22730 (22.4)	570 (22.4)
1996	172930 (20.8)	14670 (20.8)	65820 (21.4)	1930 (21.4)	46590 (20.7)	1280 (20.7)	22050 (21.7)	560 (21.7)
Maternal place of residence								
Urban	652130 (78.4)	55320 (78.4)	236950 (77.1)	6930 (77.1)	174490 (77.6)	4800 (77.6)	236950 (77.1)	6930 (77.1)
Rural	179990 (21.6)	15270 (21.6)	70570 (22.9)	2060 (22.9)	50450 (22.4)	1390 (22.4)	70570 (22.9)	2060 (22.9)
Family income quintile at birth								
Q1 (lowest)	181870 (21.9)	15430 (21.9)	67490 (21.9)	1970 (21.9)	51280 (22.8)	1410 (22.8)	67490 (21.9)	1970 (21.9)

© 2024 Ahmed A et al. *JAMA Network Open.*

Q2	171160 (20.6)	14520 (20.6)	62370 (20.3)	1820 (20.3)	45180 (20.1)	1240 (20.1)	62370 (20.3)	1820 (20.3)
Q3	161300 (19.4)	13680 (19.4)	57240 (18.6)	1670 (18.6)	42270 (18.8)	1160 (18.8)	57240 (18.6)	1670 (18.6)
Q4	158040 (19.0)	13410 (19.0)	60550 (19.7)	1770 (19.7)	43470 (19.3)	1200 (19.3)	60550 (19.7)	1770 (19.7)
Q5 (highest)	159750 (19.2)	13550 (19.2)	59870 (19.5)	1750 (19.5)	42740 (19.0)	1180 (19.0)	59870 (19.5)	1750 (19.5)

Note: All numbers were rounded to the nearest ten for confidentiality reasons

[a] Maternal age 35-39 and ≥40 categories were combined due to small cell count

[b] North America and other categories were combined due to small cell count

[c] North America and Africa were combined due to small cell count

[d] Atlantic provinces and territories were combined due to small cell count

© 2024 Ahmed A et al. *JAMA Network Open.*

eTable 4. The Transition Matrix of Probabilities That the Individual's Income Observed in a Specific Quintile at the End of Follow-Up, Conditional on Their Parents' Quintile at Baseline in the Matched Cohorts by PTB (and GA Subcategories)

a) **Term (37-41 weeks)**

	Child income quintile				
family income quintile	Q1	Q2	Q3	Q4	Q5
Q1	0.30 (0.29, 0.30)	0.22 (0.22, 0.23)	0.19 (0.19, 0.19)	0.16 (0.16, 0.16)	0.13 (0.13, 0.13)
Q2	0.22 (0.21, 0.22)	0.20 (0.20, 0.21)	0.21 (0.20, 0.21)	0.20 (0.19, 0.20)	0.18 (0.17, 0.18)
Q3	0.19 (0.18, 0.19)	0.20 (0.19, 0.20)	0.20 (0.20, 0.21)	0.21 (0.2, 0.21)	0.21 (0.20, 0.21)
Q4	0.18 (0.18, 0.18)	0.19 (0.19, 0.19)	0.19 (0.19, 0.20)	0.21 (0.21, 0.21)	0.23 (0.22, 0.23)
Q5	0.19 (0.19, 0.19)	0.18 (0.18, 0.18)	0.18 (0.18, 0.19)	0.20 (0.19, 0.20)	0.25 (0.25, 0.26)

b) **Preterm (24-36 weeks)**

	Child income quintile				
family income quintile	Q1	Q2	Q3	Q4	Q5
Q1	0.31 (0.31, 0.32)	0.23 (0.23, 0.24)	0.19 (0.18, 0.20)	0.15 (0.15, 0.16)	0.11 (0.11, 0.12)
Q2	0.23 (0.22, 0.24)	0.22 (0.21, 0.23)	0.21 (0.20, 0.21)	0.19 (0.18, 0.19)	0.16 (0.15, 0.16)
Q3	0.20 (0.19, 0.20)	0.21 (0.20, 0.21)	0.21 (0.20, 0.21)	0.20 (0.19, 0.20)	0.19 (0.18, 0.20)
Q4	0.19 (0.18, 0.19)	0.19 (0.19, 0.20)	0.20 (0.19, 0.20)	0.21 (0.21, 0.22)	0.21 (0.20, 0.22)
Q5	0.19 (0.19, 0.20)	0.19 (0.19, 0.20)	0.18 (0.17, 0.19)	0.18 (0.18, 0.19)	0.25 (0.24, 0.26)

c) **Late preterm (34-36 weeks)**

	Child income quintile				
family income quintile	Q1	Q2	Q3	Q4	Q5
Q1	0.31 (0.30, 0.32)	0.23 (0.22, 0.24)	0.19 (0.19, 0.2)	0.15 (0.15, 0.16)	0.12 (0.11, 0.12)
Q2	0.23 (0.22, 0.23)	0.22 (0.21, 0.22)	0.21 (0.2, 0.22)	0.19 (0.18, 0.20)	0.16 (0.15, 0.17)
Q3	0.19 (0.19, 0.20)	0.20 (0.19, 0.21)	0.21 (0.2, 0.22)	0.20 (0.19, 0.20)	0.20 (0.19, 0.20)
Q4	0.18 (0.18, 0.19)	0.19 (0.18, 0.20)	0.2 (0.19, 0.20)	0.21 (0.21, 0.22)	0.21 (0.21, 0.22)
Q5	0.19 (0.18, 0.19)	0.19 (0.18, 0.20)	0.18 (0.17, 0.19)	0.19 (0.18, 0.19)	0.26 (0.25, 0.27)

© 2024 Ahmed A et al. *JAMA Network Open.*

d) **Moderate preterm (32-33)**

	Child income quintile				
family income quintile	Q1	Q2	Q3	Q4	Q5
Q1	0.33 (0.30, 0.35)	0.23 (0.21, 0.25)	0.17 (0.16, 0.19)	0.15 (0.14, 0.17)	0.12 (0.10, 0.13)
Q2	0.23 (0.21, 0.25)	0.23 (0.21, 0.25)	0.20 (0.18, 0.23)	0.19 (0.17, 0.21)	0.15 (0.13, 0.17)
Q3	0.21 (0.19, 0.23)	0.21 (0.19, 0.23)	0.21 (0.19, 0.23)	0.20 (0.18, 0.22)	0.18 (0.16, 0.20)
Q4	0.19 (0.17, 0.21)	0.18 (0.16, 0.20)	0.20 (0.18, 0.22)	0.21 (0.19, 0.23)	0.22 (0.20, 0.24)
Q5	0.21 (0.19, 0.23)	0.19 (0.17, 0.21)	0.17 (0.15, 0.19)	0.18 (0.16, 0.20)	0.25 (0.23, 0.27)

e) **Very preterm (28-31 weeks)**

	Child income quintile				
family income quintile	Q1	Q2	Q3	Q4	Q5
Q1	0.33 (0.30, 0.35)	0.24 (0.21, 0.26)	0.20 (0.18, 0.22)	0.15 (0.13, 0.17)	0.09 (0.07, 0.11)
Q2	0.25 (0.22, 0.27)	0.25 (0.22, 0.28)	0.20 (0.18, 0.22)	0.17 (0.14, 0.19)	0.13 (0.11, 0.16)
Q3	0.19 (0.17, 0.22)	0.24 (0.21, 0.26)	0.21 (0.18, 0.23)	0.21 (0.19, 0.24)	0.15 (0.13, 0.18)
Q4	0.20 (0.17, 0.22)	0.22 (0.20, 0.25)	0.20 (0.18, 0.23)	0.20 (0.17, 0.22)	0.18 (0.16, 0.21)
Q5	0.21 (0.19, 0.24)	0.22 (0.19, 0.24)	0.2 (0.18, 0.23)	0.16 (0.14, 0.19)	0.20 (0.18, 0.23)

f) **Extremely preterm (24-27 weeks)**

	Child income quintile				
family income quintile	Q1	Q2	Q3	Q4	Q5
Q1	0.41 (0.36, 0.46)	0.26 (0.22, 0.30)	0.16 (0.13, 0.20)	0.09 (0.07, 0.12)	0.07 (0.05, 0.10)
Q2	0.29 (0.24, 0.35)	0.28 (0.23, 0.33)	0.18 (0.15, 0.22)	0.16 (0.12, 0.19)	0.09 (0.06, 0.12)
Q3	0.28 (0.23, 0.33)	0.28 (0.23, 0.32)	0.21 (0.17, 0.26)	0.13 (0.10, 0.17)	0.10 (0.07, 0.13)
Q4	0.26 (0.22, 0.31)	0.27 (0.22, 0.32)	0.20 (0.16, 0.25)	0.16 (0.12, 0.20)	0.10 (0.07, 0.13)
Q5	0.22 (0.17, 0.27)	0.23 (0.19, 0.28)	0.18 (0.14, 0.23)	0.20 (0.15, 0.24)	0.17 (0.12, 0.21)

© 2024 Ahmed A et al. *JAMA Network Open.*

eTable 5. Coefficients and Standard Errors for the Association Between Gestational Age Categories and Annual Income in the Matched Cohort, in Analyses Including Cross Product Terms Between Gestational Age Categories (GA) and Family Income Quintiles

a) Mean income differences

	34-36		32-33		28-31		24-27	
Variable	Coefficient	SE	Coefficient	SE	Coefficient	SE	Coefficient	SE
GA	-617	107	-949	306	-1700	324	-3810	514
Q2	2433	78	2554	219	2877	232	1952	346
Q3	3858	79	3872	209	4293	230	4091	410
Q4	4471	79	4620	201	4910	266	4023	401
Q5	6112	97	6262	236	6163	259	5355	437
GA*Q2	36	155	133	467	-409	495	41	729
GA*Q3	97	161	-190	463	-172	511	-1106	834
GA*Q4	319	165	283	466	182	564	-1391	808
GA*Q5	362	186	111	539	-880	562	280	1034

b) log-income

	34-36		32-33		28-31		24-27	
Variable	Coefficient	SE	Coefficient	SE	Coefficient	SE	Coefficient	SE
GA	-0.04	0.01	-0.05	0.02	-0.10	0.03	-0.23	0.04
Q2	0.13	0.01	0.13	0.02	0.16	0.02	0.09	0.03
Q3	0.19	0.01	0.20	0.01	0.22	0.02	0.19	0.03
Q4	0.23	0.01	0.25	0.01	0.25	0.02	0.17	0.03
Q5	0.28	0.01	0.29	0.01	0.30	0.02	0.21	0.03
GA*Q2	0.01	0.01	-0.01	0.03	-0.03	0.04	0.00	0.06
GA*Q3	0.01	0.01	0.01	0.03	0.00	0.03	0.01	0.07
GA*Q4	0.02	0.01	0.02	0.03	0.00	0.04	-0.05	0.06
GA*Q5	0.04	0.01	0.03	0.03	-0.02	0.04	0.12	0.07

© 2024 Ahmed A et al. *JAMA Network Open.*

c) percentile rank change

	34-36		32-33		28-31		24-27	
Variable	Coefficient	SE	Coefficient	SE	Coefficient	SE	Coefficient	SE
GA	-1.38	0.28	-2.32	0.78	-3.29	0.91	-8.97	1.47
Q2	-13.17	0.20	-13.29	0.52	-12.76	0.61	-13.95	0.99
Q3	-30.15	0.21	-30.14	0.53	-29.28	0.62	-30.52	0.91
Q4	-48.85	0.19	-48.60	0.50	-48.00	0.63	-49.90	0.98
Q5	-67.97	0.19	-68.22	0.52	-68.05	0.59	-70.19	0.99
GA*Q2	0.30	0.40	0.45	1.13	-1.66	1.30	-0.39	2.15
GA*Q3	0.63	0.41	-0.37	1.16	-0.36	1.32	-2.76	2.13
GA*Q4	1.01	0.42	1.05	1.16	0.08	1.35	-2.16	2.17
GA*Q5	1.56	0.41	0.94	1.15	-0.39	1.33	4.38	2.34

© 2024 Ahmed A et al. *JAMA Network Open.*

eTable 6. Associations Between Preterm Birth and Annual Family Income at or After the Age of 18 Years for Individuals Born in 1990-1996 in Canada, Overall and Stratified by Family Income Quintiles at Baseline

a) Mean annual employment income.

	Unmatched cohort- Mean income differences in CAD (95% CI) [a]					
Category	Overall	Quintile 1 (lowest)	Quintile 2	Quintile 3	Quintile 4	Quintile 5 (highest)
Preterm birth						
Preterm (<37 weeks)	-6224 (-6635, -5812)	-3655 (-3987, -3324)	-4088 (-4510, -3666)	-4489 (-5008, -3969)	-3678 (-4510, -2845)	-3879 (-5630, -2128)
Gestational age category						
34-36 weeks	-5785 (-6239, -5330)	-3248 (-3624, -2873)	-3734 (-4208, -3260)	-4171 (-4743, -3599)	-3507 (-4295, -2718)	-3027 (-5016, -1037)
32-33 weeks	-5942 (-7342, -4543)	-4913 (-5776, -4050)	-3814 (-5115, -2513)	-5376 (-6973, -3779)	-1337 (-5961, 3287)	-3915 (-8741, 911)
28-31 weeks	-9190 (-10392, -7988)	-4719 (-5796, -3643)	-6570 (-7901, -5240)	-5832 (-7676, -3989)	-7134 (-9236, -5032)	-9450 (-14512, -4387)
24-27 weeks	-13011 (-14803, -11218)	-7936 (-9610, -6261)	-9040 (-11096, -6984)	-7424 (-10962, -3886)	-9354 (-12743, -5964)	-16426 (-23692, -9159)
	Matched cohort model 1- Mean income differences in CAD (95% CI) [b]					
Category	Overall	Quintile 1 (lowest)	Quintile 2	Quintile 3	Quintile 4	Quintile 5 (highest)
Preterm birth						
Preterm (<37 w)	-2336 (-2830, -1841)	-2500 (-2925, -2075)	-2261 (-2789, -1734)	-2843 (-3486, -2199)	-1838 (-2687, -989)	-2249 (-4281, -216)
Gestational age category						
34-36 weeks	-1873 (-2422, -1323)	-2281 (-2760, -1803)	-2035 (-2615, -1456)	-2544 (-3250, -1837)	-1336 (-2263, -408)	-1127 (-3396, 1142)
32-33 weeks	-2250 (-3760, -740)	-3041 (-4278, -1803)	-1336 (-3105, 434)	-2885 (-4932, -839)	-1547 (-4457, 1363)	-2805 (-8640, 3031)
28-31 weeks	-6069 (-7575, -4563)	-3076 (-4510, -1641)	-4714 (-6434, -2994)	-5342 (-7417, -3266)	-6223 (-8975, -3472)	-11558 (-17408, -5708)
24-27 weeks	-8557 (-10966, -6149)	-6097 (-8426, -3769)	-7249 (-10016, -4481)	-6359 (-11169, -1548)	-7448 (-12224, -2671)	-13761 (-23132, -4390)

© 2024 Ahmed A et al. *JAMA Network Open.*

	Matched cohort model 2- Mean income differences in CAD (95% CI) [b, c]					
Category	Overall	Quintile 1 (lowest)	Quintile 2	Quintile 3	Quintile 4	Quintile 5 (highest)
Preterm birth<br>Preterm (<37 w)	-2333 (-2827, -1839)	-2498 (-2922, -2074)	-2257 (-2784, -1730)	-2840 (-3483, -2196)	-1837 (-2686, -988)	-2258 (-4290, -225)
Gestational age category<br>34-36 weeks<br>32-33 weeks<br>28-31 weeks<br>24-27 weeks	-1872 (-2421, -1322)<br>-2251 (-3761, -741)<br>-6070 (-7575, -4564)<br>-8529 (-10941, -6117)	-2280 (-2758, -1803)<br>-3038 (-4275, -1801)<br>-3079 (-4510, -1648)<br>-6048 (-8371, -3724)	-2034 (-2613, -1455)<br>-1329 (-3097, 439)<br>-4703 (-6422, -2984)<br>-7152 (-9905, -4400)	-2544 (-3250, -1837)<br>-2878 (-4925, -831)<br>-5322 (-7397, -3246)<br>-6222 (-11000, -1444)	-1335 (-2263, -408)<br>-1545 (-4455, 1364)<br>-6237 (-8992, -3482)<br>-7457 (-12234, -2681)	-1130 (-3399, 1139)<br>-2830 (-8655, 2995)<br>-11564 (-17409, -5719)<br>-14152 (-23571, -4733)

b) Ratio of income per year (log-income)

	Unmatched cohort- Ratio of income (95% CI) [a]					
Category	Overall	Quintile 1 (lowest)	Quintile 2	Quintile 3	Quintile 4	Quintile 5 (highest)
Preterm birth<br>Preterm (<37 w)	0.90 (0.89, 0.91)	0.9 (0.89, 0.91)	0.91 (0.9, 0.92)	0.92 (0.91, 0.93)	0.95 (0.93, 0.96)	0.96 (0.94, 0.98)
Gestational age category<br>34-36 weeks<br>32-33 weeks<br>28-31 weeks<br>24-27 weeks	0.91 (0.90, 0.92)<br>0.91 (0.88, 0.93)<br>0.85 (0.83, 0.87)<br>0.79 (0.76, 0.82)	0.91 (0.90, 0.92)<br>0.86 (0.84, 0.89)<br>0.87 (0.84, 0.90)<br>0.78 (0.73, 0.82)	0.92 (0.91, 0.93)<br>0.92 (0.89, 0.95)<br>0.86 (0.83, 0.89)<br>0.81 (0.77, 0.85)	0.93 (0.92, 0.94)<br>0.91 (0.88, 0.94)<br>0.90 (0.87, 0.93)<br>0.87 (0.81, 0.94)	0.95 (0.94, 0.96)<br>0.98 (0.92, 1.05)<br>0.90 (0.87, 0.93)<br>0.86 (0.82, 0.92)	0.97 (0.95, 0.99)<br>0.96 (0.92, 1.01)<br>0.91 (0.86, 0.96)<br>0.84 (0.77, 0.91)
	Matched cohort model 1- Ratio of income (95% CI) [b]					
Category	Overall	Quintile 1 (lowest)	Quintile 2	Quintile 3	Quintile 4	Quintile 5 (highest)
Preterm birth<br>Preterm (<37 w)	0.96 (0.95, 0.97)	0.93 (0.91, 0.94)	0.95 (0.94, 0.96)	0.95 (0.94, 0.96)	0.97 (0.96, 0.99)	0.98 (0.96, 1.00)
Gestational age category<br>34-36 weeks<br>32-33 weeks<br>28-31 weeks<br>24-27 weeks	0.97 (0.96, 0.98)<br>0.96 (0.94, 0.99)<br>0.90 (0.87, 0.92)<br>0.85 (0.82, 0.90)	0.93 (0.92, 0.95)<br>0.91 (0.88, 0.95)<br>0.91 (0.87, 0.95)<br>0.82 (0.76, 0.89)	0.96 (0.94, 0.97)<br>0.97 (0.93, 1.01)<br>0.90 (0.86, 0.93)<br>0.84 (0.78, 0.9)	0.95 (0.94, 0.97)<br>0.95 (0.91, 0.99)<br>0.90 (0.87, 0.94)<br>0.89 (0.81, 0.97)	0.98 (0.97, 0.99)<br>0.98 (0.94, 1.02)<br>0.91 (0.87, 0.95)<br>0.89 (0.83, 0.96)	0.99 (0.97, 1.01)<br>0.97 (0.92, 1.03)<br>0.89 (0.83, 0.94)<br>0.86 (0.78, 0.96)

© 2024 Ahmed A et al. *JAMA Network Open.*

	Matched cohort model 2- Ratio of income (95% CI) [b,c]					
Category	Overall	Quintile 1 (lowest)	Quintile 2	Quintile 3	Quintile 4	Quintile 5 (highest)
Preterm birth						
Preterm (<37 w)	0.96 (0.95, 0.97)	0.94 (0.93, 0.96)	0.96 (0.94, 0.97)	0.95 (0.94, 0.96)	0.98 (0.96, 0.99)	0.98 (0.96, 1.00)
Gestational age category						
34-36 weeks	0.97 (0.96, 0.98)	0.95 (0.93, 0.97)	0.96 (0.95, 0.97)	0.96 (0.95, 0.97)	0.98 (0.97, 0.99)	0.99 (0.97, 1.01)
32-33 weeks	0.96 (0.94, 0.99)	0.93 (0.89, 0.97)	0.97 (0.93, 1.02)	0.96 (0.92, 0.99)	0.99 (0.94, 1.03)	0.97 (0.91, 1.03)
28-31 weeks	0.91 (0.88, 0.93)	0.91 (0.87, 0.95)	0.91 (0.87, 0.95)	0.90 (0.87, 0.94)	0.91 (0.88, 0.96)	0.89 (0.83, 0.95)
24-27 weeks	0.87 (0.83, 0.91)	0.85 (0.78, 0.92)	0.85 (0.79, 0.93)	0.89 (0.81, 0.98)	0.89 (0.83, 0.96)	0.87 (0.78, 0.97)

[a] n= 1,614,150 individuals; 11,727,110 person-year

[b] n= 968,550 for <37 category (6,966,590 person-years); n= 897,970 for 34-36 category (6,465,520 person-years); n= 315,050 for 32-33 category (2,207,450 person-years); n= 229,770 for 28-31 category (1,611,600 person-years); n= 103,160 for 24-27 category (712,350 person-years).

[c] Model 2 used the matched sample and further adjusted for the calendar year and age modeled using restricted cubic splines.

© 2024 Ahmed A et al. *JAMA Network Open.*

eTable 7. Associations Between Preterm Birth and Mean Difference in Percentile Rank Change (Calculated Using Family Income) for Individuals Born in 1990-1996 in Canada

	Mean differences (95% CI)	
Category	Unmatched Cohorts [a]	Matched Cohorts [b]
Preterm birth		
Preterm (<37 weeks)	0.43 (0.23, 0.63)	-1.14 (-1.39, -0.89)
Gestational age category		
34-36 weeks	0.69 (0.46, 0.91)	-0.94 (-1.23, -0.66)
32-33 weeks	-0.15 (-0.74, 0.44)	-1.27 (-2.02, -0.52)
28-31 weeks	-0.76 (-1.52, 0.00)	-2.47 (-3.45, -1.50)
24-27 weeks	-1.58 (-2.83, -0.33)	-3.47 (-5.07, -1.87)

[a] n= 1,614,150 individuals

[b] n= 968,550 for <37 category; n= 897,970 for 34-36 category; n= 315,050 for 32-33 category; n= 229,770 for 28-31 category; n= 103,160 for 24-27 category.

© 2024 Ahmed A et al. *JAMA Network Open.*

eTable 8. Associations Between Preterm Birth and Annual Income at or After the Age of 18 Years When Individuals Who Died Were Assigned Zero Income, Overall and Stratified by Family Income Quintiles at Baseline

a) Mean annual employment income.

	Crude Model- Mean income differences in CAD (95% CI) [a]					
Category	Overall	Quintile 1 (lowest)	Quintile 2	Quintile 3	Quintile 4	Quintile 5 (highest)
Preterm birth						
Preterm (<37 w)	-1982 (-2066, -1898)	-1912 (-2062, -1762)	-2193 (-2360, -2025)	-1784 (-1970, -1598)	-1466 (-1661, -1271)	-1306 (-1539, -1073)
Gestational age category	-1487 (-1581, -1393)	-1547 (-1717, -1378)	-1733 (-1920, -1547)	-1310 (-1517, -1104)	-920 (-1137, -702)	-654 (-913, -396)
34-36 weeks	-2106 (-2356, -1856)	-1970 (-2407, -1534)	-2289 (-2796, -1782)	-2216 (-2767, -1664)	-1496 (-2073, -919)	-1393 (-2107, -680)
32-33 weeks	-3760 (-4039, -3482)	-3196 (-3676, -2715)	-3738 (-4316, -3159)	-3120 (-3753, -2487)	-3236 (-3914, -2558)	-4307 (-5052, -3562)
28-31 weeks	-9474 (-9861, -9087)	-7465 (-8142, -6787)	-9020 (-9770, -8271)	-9529 (-10412, -8646)	-10112 (-11036, -9189)	-10497 (-11644, -9349)
24-27 weeks						
	Matched cohort model 1- Mean income differences (95% CI) [b]					
Category	Overall	Quintile 1 (lowest)	Quintile 2	Quintile 3	Quintile 4	Quintile 5 (highest)
Preterm birth						
Preterm (<37 w)	-1063 (-1168, -959)	-1157 (-1353, -961)	-1088 (-1295, -880)	-1135 (-1360, -909)	-936 (-1171, -701)	-986 (-1271, -700)
Gestational age category	-595 (-709, -481)	-770 (-988, -551)	-668 (-896, -440)	-646 (-893, -400)	-436 (-694, -179)	-425 (-732, -119)
34-36 weeks	-1320 (-1649, -991)	-1354 (-1957, -752)	-1210 (-1885, -536)	-1646 (-2356, -937)	-1153 (-1869, -437)	-1257 (-2199, -315)
32-33 weeks	-3039 (-3399, -2679)	-2681 (-3324, -2038)	-2947 (-3694, -2200)	-2992 (-3799, -2185)	-2791 (-3683, -1899)	-3866 (-4791, -2942)
28-31 weeks	-8329 (-8861, -7796)	-6961 (-7937, -5986)	-7476 (-8475, -6476)	-8931 (-10189, -7674)	-9331 (-10507, -8154)	-9452 (-11047, -7857)
24-27 weeks						
	Matched cohort model 2- Mean income differences (95% CI) [b, c]					
Category	Overall	Quintile 1 (lowest)	Quintile 2	Quintile 3	Quintile 4	Quintile 5 (highest)
Preterm birth						
Preterm (<37 w)	-1062 (-1164, -961)	-1159 (-1350, -968)	-1084 (-1284, -883)	-1132 (-1350, -913)	-935 (-1162, -707)	-984 (-1263, -705)
Gestational age category	-595 (-706, -485)	-772 (-984, -560)	-666 (-885, -446)	-644 (-882, -406)	-436 (-684, -187)	-424 (-723, -125)
34-36 weeks	-1320 (-1642, -997)	-1363 (-1955, -770)	-1200 (-1860, -541)	-1650 (-2331, -970)	-1153 (-1847, -459)	-1254 (-2185, -323)
32-33 weeks	-3028 (-3384, -2673)	-2672 (-3302, -2042)	-2933 (-3670, -2197)	-2977 (-3771, -2184)	-2777 (-3664, -1890)	-3861 (-4768, -2955)
28-31 weeks	-8320 (-8852, -7787)	-6961 (-7925, -5997)	-7460 (-8463, -6458)	-8913 (-10175, -7651)	-9312 (-10489, -8136)	-9443 (-11043, -7842)
24-27 weeks						

© 2024 Ahmed A et al. *JAMA Network Open.*

b) Ratio of income per year

	Crude Model- Ratio of income (95% CI) [a]					
Category	Overall	Quintile 1 (lowest)	Quintile 2	Quintile 3	Quintile 4	Quintile 5 (highest)
Preterm birth						
Preterm (<37 weeks)	0.90 (0.9, 0.91)	0.89 (0.88, 0.9)	0.89 (0.88, 0.9)	0.91 (0.91, 0.92)	0.93 (0.92, 0.94)	0.94 (0.93, 0.95)
Gestational age category						
34-36 weeks	0.93 (0.92, 0.93)	0.91 (0.90, 0.92)	0.91 (0.90, 0.92)	0.94 (0.93, 0.95)	0.96 (0.95, 0.97)	0.97 (0.96, 0.98)
32-33 weeks	0.90 (0.89, 0.91)	0.89 (0.86, 0.91)	0.88 (0.86, 0.91)	0.89 (0.87, 0.92)	0.93 (0.90, 0.96)	0.94 (0.91, 0.97)
28-31 weeks	0.82 (0.80, 0.83)	0.81 (0.79, 0.84)	0.81 (0.78, 0.84)	0.85 (0.82, 0.88)	0.85 (0.82, 0.88)	0.81 (0.78, 0.85)
24-27 weeks	0.54 (0.52, 0.56)	0.57 (0.53, 0.61)	0.55 (0.51, 0.59)	0.55 (0.51, 0.59)	0.53 (0.49, 0.57)	0.54 (0.50, 0.60)
	Adjusted Model 1- Ratio of income (95% CI) [b]					
Category	Overall	Quintile 1 (lowest)	Quintile 2	Quintile 3	Quintile 4	Quintile 5 (highest)
Preterm birth						
Preterm (<37 w)	0.95 (0.94, 0.95)	0.93 (0.92, 0.94)	0.94 (0.93, 0.95)	0.94 (0.93, 0.96)	0.96 (0.94, 0.97)	0.96 (0.94, 0.97)
Gestational age category						
34-36 weeks	0.97 (0.96, 0.98)	0.95 (0.94, 0.97)	0.96 (0.95, 0.98)	0.97 (0.96, 0.98)	0.98 (0.97, 0.99)	0.98 (0.97, 0.99)
32-33 weeks	0.93 (0.92, 0.95)	0.92 (0.88, 0.95)	0.94 (0.90, 0.97)	0.92 (0.89, 0.95)	0.95 (0.91, 0.98)	0.94 (0.91, 0.99)
28-31 weeks	0.85 (0.83, 0.87)	0.84 (0.80, 0.88)	0.85 (0.81, 0.89)	0.86 (0.82, 0.89)	0.87 (0.83, 0.91)	0.83 (0.79, 0.87)
24-27 weeks	0.58 (0.55, 0.60)	0.59 (0.54, 0.64)	0.60 (0.56, 0.65)	0.57 (0.52, 0.63)	0.55 (0.50, 0.60)	0.58 (0.52, 0.65)
	Adjusted Model 2- Ratio of income (95% CI) [b, c]					
Category	Overall	Quintile 1 (lowest)	Quintile 2	Quintile 3	Quintile 4	Quintile 5 (highest)
Preterm birth						
Preterm (<37 w)	0.95 (0.94, 0.95)	0.93 (0.92, 0.94)	0.94 (0.93, 0.95)	0.94 (0.93, 0.96)	0.95 (0.94, 0.97)	0.96 (0.95, 0.98)
Gestational age category						
34-36 weeks	0.97 (0.96, 0.98)	0.95 (0.93, 0.97)	0.96 (0.95, 0.98)	0.97 (0.95, 0.98)	0.98 (0.96, 0.99)	0.99 (0.97, 1.00)
32-33 weeks	0.94 (0.92, 0.95)	0.93 (0.88, 0.97)	0.91 (0.87, 0.96)	0.93 (0.89, 0.98)	0.94 (0.90, 0.99)	0.96 (0.91, 1.01)
28-31 weeks	0.84 (0.82, 0.86)	0.85 (0.80, 0.90)	0.83 (0.79, 0.87)	0.84 (0.80, 0.89)	0.84 (0.79, 0.88)	0.82 (0.78, 0.87)
24-27 weeks	0.58 (0.55, 0.61)	0.6 (0.54, 0.66)	0.59 (0.53, 0.65)	0.58 (0.51, 0.65)	0.55 (0.49, 0.61)	0.58 (0.51, 0.66)

[a] n= 1,614,150 individuals; 11,727,110 person-year

[b] n= 968,550 for <37 category (6,966,590 person-years); n= 897,970 for 34-36 category (6,465,520 person-years); n= 315,050 for 32-33 category (2,207,450 person-years); n= 229,770 for 28-31 category (1,611,600 person-years); n= 103,160 for 24-27 category (712,350 person-years).

[c] Model 2 used the matched sample and further adjusted for the calendar year and age modeled using restricted cubic splines.

© 2024 Ahmed A et al. *JAMA Network Open.*

eTable 9. Associations Between Preterm Birth and Mean Difference in Percentile Rank Change When Individuals Who Died Were Assigned Lowest Percentile

	Mean differences (95% CI)	
Category	Unmatched cohort [a]	Matched cohorts [b]
Preterm birth		
Preterm (<37 weeks)	0.09 (-0.15, 0.34)	-2.12 (-2.42, -1.83)
Gestational age category		
34-36 weeks	1.22 (0.94, 1.50)	-1.03 (-1.36, -0.70)
32-33 weeks	-0.62 (-1.31, 0.07)	-2.94 (-3.83, -2.06)
28-31 weeks	-3.39 (-4.22, -2.56)	-6.38 (-7.46, -5.29)
24-27 weeks	-16.85 (-18.15, -15.56)	-19.04 (-20.75, -17.34)

[a] n= 1,614,150 individuals

[b] n= 968,550 for <37 category; n= 897,970 for 34-36 category; n= 315,050 for 32-33 category; n= 229,770 for 28-31 category; n= 103,160 for 24-27 category.

© 2024 Ahmed A et al. *JAMA Network Open.*

eTable 10. Associations Between Preterm Birth and Annual Income at or After the Age of 18 Years, Overall and Stratified by Family Income Quintiles at Baseline, Only Among Singletons

a) Mean annual employment income.

Category	Unmatched cohorts	Matched cohort
Preterm birth		
Preterm (<37 w)	-1666 (-1755, -1578)	-667 (-771, -563)
Gestational age category		
34-36 weeks	-1446 (-1544, -1349)	-443 (-557, -329)
32-33 weeks	-1761 (-2043, -1479)	-810 (-1141, -480)
28-31 weeks	-2962 (-3270, -2654)	-2081 (-2444, -1717)
24-27 weeks	-5452 (-5936, -4968)	-4253 (-4833, -3673)

Note: analyses stratified by family income quintile were not included because of data confidentiality and security reasons (small count differences between the original cohort and the cohort restricted to singletons)

b) Ratio of income per year

	Crude Model- Ratio of income (95% CI)					
Category	Overall	Quintile 1 (lowest)	Quintile 2	Quintile 3	Quintile 4	Quintile 5 (highest)
Preterm birth						
Preterm (<37 weeks)	0.92 (0.91, 0.92)	0.90 (0.89, 0.91)	0.90 (0.89, 0.91)	0.93 (0.92, 0.94)	0.95 (0.94, 0.96)	0.97 (0.96, 0.98)
Gestational age category						
34-36 weeks	0.93 (0.92, 0.93)	0.91 (0.90, 0.92)	0.91 (0.90, 0.92)	0.94 (0.93, 0.95)	0.96 (0.95, 0.97)	0.98 (0.97, 0.99)
32-33 weeks	0.91 (0.90, 0.93)	0.90 (0.87, 0.93)	0.90 (0.87, 0.93)	0.91 (0.88, 0.95)	0.96 (0.93, 0.99)	0.95 (0.90, 0.99)
28-31 weeks	0.85 (0.83, 0.86)	0.85 (0.81, 0.88)	0.83 (0.79, 0.87)	0.88 (0.85, 0.92)	0.91 (0.68, 0.94)	0.85 (0.81, 0.90)
24-27 weeks	0.69 (0.66, 0.73)	0.73 (0.66, 0.78)	0.71 (0.65, 0.77)	0.71 (0.64, 0.78)	0.67 (0.60, 0.74)	0.78 (0.69, 0.88)
	Adjusted Model - Ratio of income (95% CI)					
Category	Overall	Quintile 1 (lowest)	Quintile 2	Quintile 3	Quintile 4	Quintile 5 (highest)
Preterm birth						
Preterm (<37 w)	0.97 (0.96, 0.97)	0.95 (0.94, 0.96)	0.96 (0.95, 0.97)	0.97 (0.95, 0.98)	0.97 (0.96, 0.98)	0.98 (0.97, 0.99)
Gestational age category						
34-36 weeks	0.98 (0.97, 0.98)	0.96 (0.95, 0.98)	0.97 (0.96, 0.98)	0.98 (0.96, 0.99)	0.99 (0.97, 1.00)	0.99 (0.98, 1.00)
32-33 weeks	0.96 (0.94, 0.98)	0.95 (0.91, 0.98)	0.96 (0.93, 1.00)	0.95 (0.91, 0.99)	0.97 (0.93, 1.00)	0.96 (0.92, 1.00)
28-31 weeks	0.89 (0.87, 0.91)	0.90 (0.85, 0.94)	0.88 (0.84, 0.92)	0.90 (0.86, 0.94)	0.92 (0.87, 0.96)	0.86 (0.82, 0.91)
24-27 weeks	0.76 (0.72, 0.80)	0.75 (0.97, 0.82)	0.77 (0.71, 0.84)	0.74 (0.66, 0.82)	0.72 (0.64, 0.80)	0.83 (0.72, 0.94)

© 2024 Ahmed A et al. *JAMA Network Open.*

eTable 11. Associations Between Preterm Birth and Mean Difference in Percentile Rank Change Only Among Singletons

	Mean differences (95% CI)	
Category	Unmatched cohort [a]	Matched cohorts [b]
Preterm birth		
Preterm (<37 weeks)	1.27 (1.02, 1.52)	-1.23 (-1.53, -0.94)
Gestational age category		
34-36 weeks	1.60 (1.32, 1.89)	-0.75 (-1.07, -0.42)
32-33 weeks	0.84 (0.054, 1.64)	-1.87 (-2.78, -0.96)
28-31 weeks	-0.47 (-1.43, 0.49	-3.76 (-4.88, -2.64)
24-27 weeks	-3.97 (-5.55, -2.40)	-8.40 (-10.29, -6.50)

© 2024 Ahmed A et al. *JAMA Network Open.*

eTable 12. Associations Between Preterm Birth and Annual Income at or After the Age of 18 Years for Individuals Born in 1990-1996 in Canada, Stratified by Sex

	Mean annual employment income			
	Females		Males	
Category	Unmatched cohorts	Matched cohorts	Unmatched cohorts	Matched cohorts
Preterm (<37 w)	-1500 (-1632, -1449)	-680 (-789, -562)	-1900 (-2011, -1749)	-730 (-888, -564)
GA category				
34-36 weeks	-1300 (-1379, -1175)	-440 (-559, -310)	-1700 (-1826, -1533)	-480 (-662, -305)
32-33 weeks	-2000 (-2232, -1676)	-1140 (-1512, -774)	-1700 (-2120, -1341)	-690 (-1184, -191)
28-31 weeks	-2700 (-3050, -2437)	-1800 (-2227, -1425)	-3000 (-3386, -2519)	-2100 (-2702, -1580)
24-27 weeks	-4700 (-5153, -4156)	-3700 (-4403, -3080)	-6200 (-6852, -5471)	-5000 (-5901, -4083)
	Ratio of income per year			
	Females		Males	
Category	Unmatched cohorts	Matched cohorts	Unmatched cohorts	Matched cohorts
Preterm (<37 w)	0.92 (0.91, 0.92)	0.96 (0.95, 0.97)	0.92 (0.91, 0.92)	0.97 (0.96, 0.97)
GA category				
34-36 weeks	0.93 (0.92, 0.94)	0.98 (0.97, 0.98)	0.93 (0.92, 0.93)	0.98 (0.97, 0.99)
32-33 weeks	0.90 (0.88, 0.91)	0.94 (0.91, 0.95)	0.92 (0.91, 0.94)	0.97 (0.94, 0.99)
28-31 weeks	0.85 (0.84, 0.87)	0.90 (0.87, 0.91)	0.87 (0.85, 0.89)	0.90 (0.88, 0.93)
24-27 weeks	0.75 (0.72, 0.77)	0.79 (0.75, 0.83)	0.73 (0.70, 0.76)	0.77 (0.73, 0.81)

© 2024 Ahmed A et al. *JAMA Network Open.*

eTable 13. Associations Between Preterm Birth and Mean Difference in Percentile Rank Change for Individuals Born in 1990-1996 in Canada in the Matched Cohort, Stratified by Sex

	Females		Males	
Category	Mean percentile rank change (95% CI)	Mean difference in percentile rank change (95% CI)	Mean percentile rank change (95% CI)	Mean difference in percentile rank change (95% CI)
Preterm birth				
Preterm (<37 w)	-3.89 (-4.26, -3.52)	-1.24 (-1.66, -0.82)	1.50 (1.15, 1.86)	-1.23 (-1.64, -0.82)
Term (37-41 w)	-2.65 (-2.76, -2.53)	Ref.	2.73 (2.62, 2.85)	Ref.
GA category				
34-36 weeks	-3.36 (-3.78, -2.95)	-0.62 (-1.08, -0.15)	1.87 (1.47, 2.28)	-0.79 (-1.24, -0.34)
32-33 weeks	-4.91 (-6.06, -3.75)	-2.41 (-3.69, -1.12)	0.94 (-0.18, 2.05)	-1.63 (-2.85, -0.41)
28-31 weeks	-6.61 (-8.05, -5.16)	-4.18 (-5.8, -2.56)	-0.42 (-1.73, 0.88)	-3.55 (-5.00, -2.09)
24-27 weeks	-10.09 (-12.33, -7.85)	-9.14 (-11.61, -6.68)	-3.68 (-6.07, -1.29)	-7.97 (-10.58, -5.36)

© 2024 Ahmed A et al. *JAMA Network Open.*

eTable 14. Associations Between Preterm Birth and Mean Difference in Percentile Rank Change for Individuals Born in 1990-1996 in Canada in the Matched Cohort, Stratified by Family Income Quintile at Baseline

	Mean difference in percentile rank change (95% CI)				
Category	Q1	Q2	Q3	Q4	Q5
Preterm birth					
Preterm (<37 w)	-1.83 (-2.33, -1.34)	-1.56 (-2.08, -1.04)	-1.44 (-1.95, -0.93)	-0.88 (-1.41, -0.36)	-0.33 (-0.86, 0.21)
Term (37-41 w)	Ref.	Ref.	Ref.	Ref.	Ref.
GA category					
34-36 weeks	-1.37 (-1.94, -0.8)	-1.01 (-1.58, -0.44)	-0.85 (-1.42, -0.29)	-0.32 (-0.90, 0.26)	0.16 (-0.43, 0.76)
32-33 weeks	-2.38 (-3.90, -0.86)	-1.86 (-3.35, -0.37)	-2.54 (-4.09, -0.98)	-1.3 (-2.88, 0.27)	-1.49 (-3.11, 0.14)
28-31 weeks	-3.58 (-5.38, -1.78)	-4.94 (-6.77, -3.10)	-3.7 (-5.57, -1.82)	-3.45 (-5.41, -1.49)	-3.54 (-5.47, -1.60)
24-27 weeks	-9.58 (-12.36, -6.8)	-9.19 (-12.18, -6.2)	-11.57 (-14.77, -8.37)	-11.43 (-14.72, -8.14)	-4.1 (-7.76, -0.44)

© 2024 Ahmed A et al. *JAMA Network Open.*

eTable 15. Associations Between Preterm Birth and Mean Difference in Percentile Rank Change for Individuals Born in 1990-1996 in Canada in the Matched Cohort, Stratified by Age Group

	Age 22-25	Age 26-28
Category	Mean difference in percentile rank change (95% CI)	Mean difference in percentile rank change (95% CI)
Preterm (<37 w)	-1.19 (-1.52, -0.86)	-1.43 (-2.09, -0.78)
Gestational age category		
34-36 weeks	-0.65 (-1.01, -0.29)	-0.99 (-1.72, -0.27)
32-33 weeks	-1.88 (-2.86, -0.90)	-2.51 (-4.76, -0.27)
28-31 weeks	-3.63 (-4.83, -2.43)	-4.30 (-7.14, -1.46)
24-27 weeks	-8.90 (-10.87, -6.94)	-6.62 (-11.86, -1.38)

© 2024 Ahmed A et al. *JAMA Network Open.*

eTable 16. Associations Between Preterm Birth and Mean Difference in Income-z-Score Change for Individuals Born in 1990-1996 in Canada in the Matched Cohort

	Mean differences (95% CI)
Preterm (<37 weeks)	-0.03 (-0.03, -0.02)
Gestational age category	
34-36 weeks	-0.02 (-0.03, -0.01)
32-33 weeks	-0.03 (-0.05, 0.00)
28-31 weeks	-0.09 (-0.12, -0.05)
24-27 weeks	-0.20 (-0.24, -0.14)

© 2024 Ahmed A et al. *JAMA Network Open.*
