# Supplementary material for: Preterm Birth, Family Income, and Intergenerational Income Mobility
Source: JAMA Netw Open. 2024 Jun 10;7(6):e2415921. doi: 10.1001/jamanetworkopen.2024.15921 (PMC11165381; doi:10.1001/jamanetworkopen.2024.15921)
Supplement: Supplement 2. — Data Sharing Statement [file jamanetwopen-e2415921-s002.pdf]

Data Sharing Statement

Ahmed. Preterm Birth, Family Income, and Intergenerational Income Mobility. *JAMA Netw Open*. Published June 10, 2024. doi:10.1001/jamanetworkopen.2024.15921

Data
Data available: No

Additional Information
Explanation for why data not available: Due to data privacy considerations, access to the data is available only through Statistics Canada
